# Marine Plankton during the Polar Night: Environmental Predictors of Spatial Variability

**DOI:** 10.3390/biology12030368

**Published:** 2023-02-25

**Authors:** Vladimir G. Dvoretsky, Marina P. Venger, Anastasya V. Vashchenko, Veronika V. Vodopianova, Ivan A. Pastukhov, Tatyana M. Maksimovskaya

**Affiliations:** Murmansk Marine Biological Institute of the Russian Academy of Sciences (MMBI RAS), 183010 Murmansk, Russia

**Keywords:** bacterioplankton, virioplankton, chlorophyll *a*, zooplankton, nutrients, Arctic Ocean, generalized linear models, redundancy analysis

## Abstract

**Simple Summary:**

Plankton are a key component of Arctic marine ecosystems, connecting all trophic levels and being involved in organic matter recycling. Our main purpose was to reveal differences in plankton characteristics in two Arctic sites that were strongly affected by the inflow of warm Atlantic waters during the period of polar night. We detected similar bacterial abundances in both regions, while other plankton parameters were significantly different. Temperature, salinity, and sampling depth shaped the plankton communities. Phytoplankton biomass also had a marked influence on microbial abundance and zooplankton assemblages. Changes in the proportions of boreal taxa suggested the Atlantification of pelagic communities, a phenomenon reported in the Arctic due to global warming.

**Abstract:**

We studied the spatial patterns of the planktonic ecosystems at two Arctic sites strongly affected by Atlantic Inflow (FS, the Fram Strait; and BS, the Barents Sea). A high degree of similarity in the bacterial abundance (mean: 3.1 × 10^5^ cells mL^−1^ in FS vs. 3.5 × 10^5^ cells mL^−1^ in BS) was found, while other plankton characteristics were different. Bacterial biomass reached a maximum in BS (3.2–7.9 mg C m^−3^), while viral abundances tended to be higher in FS (2.0–5.7 × 10^6^ particles mL^−1^). Larger bacterial cells were found in BS, suggesting the presence of different bacterial populations at both locations. The virus-to-bacteria ratio was significantly higher in FS than in BS (13.5 vs. 4.7). Chlorophyll *a* concentration was extremely low (<0.25 mg m^−3^). The highest zooplankton abundance was in the surface layer (919 individuals m^−3^ in FS vs. 602 ind. m^−3^ in BS). Zooplankton biomass strongly varied (1–39 mg C m^−3^), with the maximum in BS. High proportions of boreal taxa in the total zooplankton abundance indicate the Atlantification of pelagic ecosystems in the Arctic. Plankton indicators are correlated with temperature, salinity, and sampling depth. Strong intercorrelations were found between major plankton groups, suggesting tight links in the studied plankton ecosystems.

## 1. Introduction

The Arctic Ocean and adjacent marginal seas have been documented to be strongly affected by climate changes observed during the past few decades [1]. The global water temperature has demonstrated an increasing trend since the 1970s that caused gradual warming in the Arctic [2,3,4,5]. Sea ice extent has rapidly decreased in the Arctic seas, and earlier ice melting and the northward retreat of ice cover have greatly impacted marine environments and biota [6]. Total net primary production (NPP) and chlorophyll *a* values may be considered to be integral indicators of marine ecosystems in the Arctic, and these were reported to have increased in the last few decades [7,8,9]. Moreover, this trend is proposed to continue in the future [10,11]. Such climatic fluctuations have led to the alteration of pelagic assemblages and food web structures in the Arctic [12,13,14,15]. The most prominent consequence is the borealization of the Arctic biota, a process widely documented in recent reports [6,16,17,18,19]. Many taxa were found to expand their ranges, and some boreal taxa were recorded in more northern regions, due to ocean warming [6,16]. The total biomass of krill and fishes has increased in the Barents Sea (BS) from the 1990s to the 2000s [15,16,20]. The boreal copepods and euphausiids have expanded northwards, while some Arctic species have declined in range and biomass, and have moved further north [9,20]. In the Fram Strait, the boreal copepod *Calanus finmarchicus* transported with Atlantic inflow began to develop and grow faster, owing to higher temperatures [21]. Zooplankton biomass was found to be negatively correlated with the temperature of the Atlantic Water, and it decreased as the abundances of small zooplankton taxa increased [5,22,23].

Plankton are a major component of any aquatic ecosystem, playing a crucial role in biogeochemical cycles in the World ocean [24,25]. In the Arctic, the phytoplankton growth season is short and is controlled by the annual cycle of light regime and nutrients, as well as by the sea ice dynamics [26,27,28]. Sea-ice melting triggers an annual spring phytoplankton outburst which is dominated mainly by diatoms [29,30]. Much of the primary production formed in the spring is sequestered in the benthos [31,32], supporting extremely rich benthic populations which are utilized by higher trophic-level taxa [27,28]. The summer stratification of the upper layer declines the vertical replenishment of surface nutrients, resulting in growing biomasses of small flagellates [29,30]. The Arctic plankton foodwebs are considered to be short (from diatoms to top predators) and very sensitive to environmental forcing [26]. Zooplankton are thought to be an important intermediate link connecting primary producers to higher trophic levels [9,24,25]. Microbial assemblages have a great contribution to the total productivity, being involved in the pelagic foodweb as producers (autotrophic microbes), consumers, and transformers of dissolved organic matter (heterotrophic protists and bacteria) [26,29,33]. Viruses also play a key role in controlling microbial populations, and they are responsible for a significant part of prokaryotic and phytoplankton mortality [34]. Virioplankton affect marine nutrient cycling and the transport of organic matter through the ‘viral shunt’ [35,36].

The Fram Strait (FS) and BS are two major pathways for Atlantic Water flowing into the Arctic Ocean. Four main water masses can be divided in BS: warm Atlantic Water (AW, temperature = 1–8 °C, salinity = 34.9–35.2 psu), cold Arctic Water (ArW, temperature = −1.8–2 °C, salinity = 32.0–34.8 psu), Coastal Water (CW, temperature = −1.8–9 °C, salinity = 32.5–34.9 psu), and Barents Sea Water (BSW, temperature = −1–5 °C, salinity = 34.5–35.0 psu) [28]. The Polar Front represents a zone separating cold and warm waters. In FS, there are two current systems: the warm West Spitsbergen Current (WSC) and the cold East Greenland Current (EGC). Water masses in the region are AW (temperature > 2 °C, salinity > 34.2 psu), cold AW (temperature = 0–2 °C, salinity > 34.2 psu), Intermediate Water (temperature < 0 °C, salinity > 34.2 psu), and ArW (temperature < 2 °C, salinity < 34.9 psu) [37,38].

BS and FS are two of the most productive regions in the Arctic [10,27], having enhanced primary production relative to other Arctic sites. The Southern BS and the Western FS support rich pelagic and benthic assemblages [38,39,40,41,42,43]. These systems have been intensively investigated since the 1900s [26,27,28,37]. Despite significant research efforts in studying plankton in BS and FS, some important issues remain poorly explored. Plankton communities exhibit clear seasonal changes associated with fluctuations in environmental variables (nutrient concentration, light intensity, grazing impact, predation pressure, and hydrological conditions). A bulk of previous studies has been focused on the spring–autumn period [44,45,46,47,48,49,50,51,52,53,54,55,56,57,58,59,60] while the winter season is less well studied due to severe environmental conditions (the presence of ice cover, low temperatures, storms, and polar night). Recent publications have reported the activities of pelagic communities under such extreme conditions [45,61,62,63,64,65]. To adequately evaluate the seasonal dynamics of the plankton in the Arctic, complex winter investigations focused on the main groups (from viruses to zooplankton) are strongly needed.

Here, we present a comparative analysis of the plankton collected in two Arctic regions during the period of the polar night. Our study aimed to reveal the spatial distributions of virio-, bacterio-, phyto-, and zooplankton in the Southern BS and in the Eastern FS. Considering the strong Atlantic influence at both sites, we hypothesized that the plankton abundance and biomass would demonstrate similar regional patterns. Two-way PERMANOVA was selected to test our hypothesis regarding the similarity of plankton abundances in the two Arctic regions. Another goal of our work was to evaluate the main factors driving the plankton abundance in the winter season. To date, there are no previous studies regarding marine viruses in FS during the polar night, and our study is the first report on this plankton group. Additionally, we obtain novel data regarding other major microplankton in the Arctic marine environment.

## 2. Materials and Methods

### 2.1. Sampling and Processing

**Figure 1:**
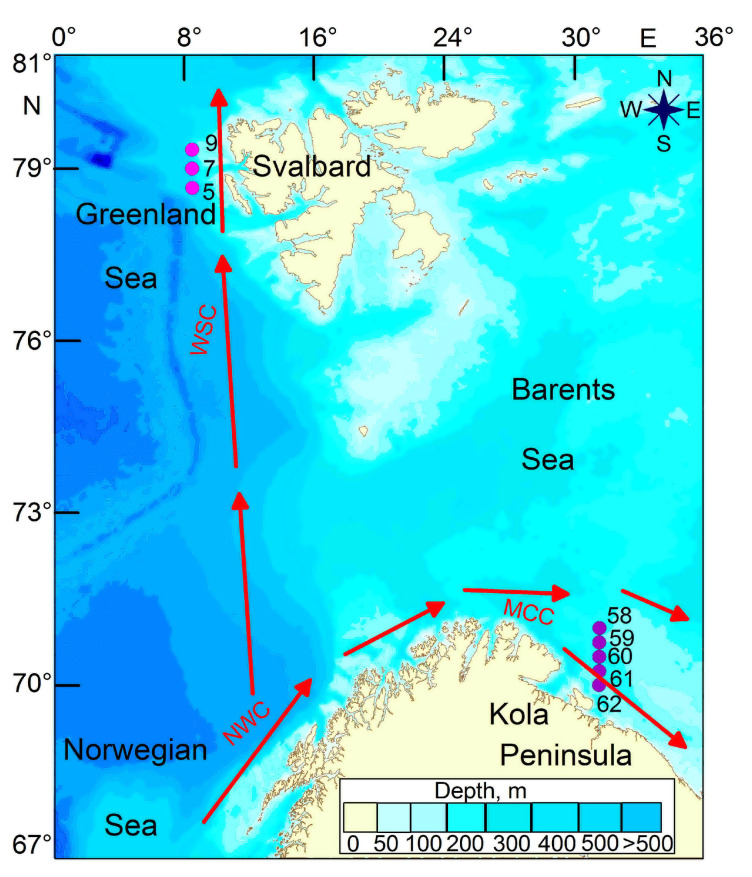
Map of sampling stations in the Fram Strait and in the Barents Sea, winter 2021. Red arrow denotes main Atlantic flows (WSC—West Spitsbergen Current, NWC—Norwegian Coastal Current, MCC—Murmansk Coastal Current).

Oceanographic data, microbial, nutrient, and zooplankton samples were collected during a multidisciplinary cruise on board the R/V Dalnie Zelentsy during the winter season (24 November to 18 December 2021) (Figure 1, Table 1). Two transects were investigated in FS (3 stations) and in BS (5 stations).

A CTD profiler, SEACAT SBE 19plus V2, was used to determine the oceanographic values and the water column structure. Sampling for microbial plankton, nutrients (nitrate, phosphate, silicate, and dissolved oxygen), and chlorophyll *a* (Chl-a) was carried out with 10 L Niskin bottles mounted on a Rosette system.

Five to seven fixed-depth layers (0 m, 10 m, 25 m, 50 m, 100 m, 200 m, and near the bottom), or less if the bottom was shallower, were sampled. Water samples for nutrient analyses (120 samples) were immediately filtered through a 0.45 µm polycarbonate membrane (FMPA, Vladisart, Vladimir, Russia), and kept frozen in liquid nitrogen in polyethylene 20 mL bottles and stored at −20 °C until their analysis in the Murmansk Marine Biological Institute laboratory. Chl-a samples (2–5 L for each sampling stratum) were filtered through 0.6 µm Vladiopore filters and then frozen (−20 °C) until the analysis. To estimate bacterial abundance and biomass, water samples were collected into plastic vials and preserved by adding a pre-filtered formaldehyde solution to a final concentration of 2%. Bacterial samples were kept in slide boxes at −20 °C. A total of 43 and 81 samples of Chl-a and marine microbes were collected, respectively (Table 1).

After the Rosette being taken onboard, vertical zooplankton hauls were made using a 50 cm mouth diameter closing WP-2 net with a mesh size of 180 μm. The water volume filtered through the net during sampling was estimated with a calibrated flowmeter (Hydrobios). Sampling was performed at two sampling layers (0–50 m, and 50 m or near the bottom) to evaluate the variability in the total abundance in the upper and sub-surface strata. The samples were preserved using a 4% formalin solution in seawater buffered with borax. In total, 16 zooplankton samples were obtained.

### 2.2. Laboratory Procedures

The determination of nutrients was performed in accordance with a standard manual [66]. Dissolved oxygen content was determined following the Winkler method using an automatic-endpoint-detection burette, Digital Burette VITLAB (63762 Grossostheim, Germany). The precisions of the hydrochemical analyses were 0.1 μM (nitrate and silicate) and 0.04 μM (phosphate). In the laboratory, the Chl-a filters were extracted in 90% acetone, kept in a freezer at 4 °C for 24 h, and centrifuged, following a standard procedure [67]. The fluorescence was determined with a Nicolett Evolution 500 spectrophotometer (Spectronic Unicam, Scotia, NY, USA), previously calibrated by the manufacturer. Chl-a content (mg m^−3^) was used as a measure of the total phytoplankton biomass.

The microscopic analysis of bacterioplankton samples was made as follows: sub-samples were incubated with DAPI for 8–10 min and filtered through 0.2 µm Nuclepore filters at a vacuum pressure of 10–12 cm Hg [68]. The filter films were dried and then fixed to glass slides with fluorescence-free immersion oil and stored at −30 °C until analysis. An OlympusBX 53 epifluorescence microscope was used to examine the DAPI-stained samples. Approximately 400–500 bacterial cells were counted per film in 20 fields randomly selected for counting at a 1000× magnification. The bacterial abundance was expressed as the number of cells per 1 L. The dimensions of 30–50 bacterial cells in each sample were measured to calculate the average bacterial cell volume (ABV—average bacterial cell volume, µm^3^). The carbon biomass (C, fg cell^−1^) was estimated according to Norland [69].

Virioplankton samples were stained with SYBR Green I fluorochrome (Molecular Probes, Eugene, OR, USA) [70]. A volume of 0.5–1.0 mL of seawater was filtered through 0.02 μm Anodisc aluminum oxide membrane filters (Whatman). For the counting of viruses, the films were examined under an OlympusBX 53 epifluorescence microscope at a magnification of 1000×. View fields were randomly selected and enumerated until the total counts exceeded 200 particles. The ratio of viral to bacterial abundance (VBR) was calculated for each sampling layer.

The zooplankton samples were divided in the laboratory using a Folsom splitter. Sub-samples (1/16–1/32 of the total sample) were examined using an MBS-10 stereomicroscope at a magnification of 32–56×. The organisms were identified to the lowest possible taxonomical levels, in accordance with our previous studies [22,23,55,56,57,58,59,71]. Zooplankton abundance for each sampling layer was expressed as individuals m^−3^. Biomass was the calculated length–mass or the published mean individual wet, dry, or carbon masses [72,73,74,75]. All values were then presented as mg carbon mass (DM) per 1 cubic meter according to the equations: 1 mg wet mass = 0.04 mg dry mass = 0.02 mg C for gelatinous zooplankton, and 1 mg wet mass = 0.2 mg dry weight = 0.1 mg C for other taxa [76].

### 2.3. Statistical Analyses

Descriptive statistics (ranges and means with standard deviations) were calculated for the oceanographic and biological datasets. The data normality was checked with a Kolmogorov–Smirnov test, and a modified Levene’s test was used for checking the homogeneity of variances. Principal component analysis (PCA) and one-way PERMANOVA [77] were performed to identify the similarity in environmental conditions (temperature, salinity, and nutrient concentrations) between stations. The Pearson correlation was used to calculate the resemblance matrix for the environmental data in PCA [78]. Two-way PERMANOVA with two fixed factors was applied to test for differences in plankton variables (microbial abundance and biomass, AVB, VBR, Chl-a concentration, and zooplankton abundance and biomass) between the two regions and among sampling layers. Pair comparisons of each environmental or biological variable were made with one-way ANOVA or Kruskal-Wallis tests (in the case of non-normal data distribution) [78]. Descriptive statistics, PCA, PERMANOVA, ANOVA, and Kruskal-Wallis tests were performed in PAST 3.22 [79].

The zooplankton community was analyzed using multivariate techniques. The Shannon–Wiener index (H’) [80] and Pielou’s evenness (J) [81] were calculated to estimate the diversity of the zooplankton assemblages.

The zooplankton dataset was lg(x+1)-transformed, and the Bray–Curtis similarity index was calculated for all stations [82]. Hierarchical cluster analysis (group-average linkage method) was used to explore patterns in the zooplankton community structure [78]. An analysis of similarity (ANOSIM) was applied to test for differences between groups of samples. The similarity percentage (SIMPER) routine was carried out to identify the percent contribution of zooplankton taxa to the cluster. All analyses were performed using Primer version 5.2.3 software [83].

The detrended correspondence analysis (DCA) was performed to select between canonical correspondence and redundancy analyses with Canoco version 4.5.6 software [84]. Rare species (occurrence in samples < 20%) were removed from the dataset to reduce the influence of outliers before using DCA. The longest gradients of the (DCA) ordination axis were 0.541–0.945, suggesting that redundancy analysis (RDA) was suitable for investigating the relationship between zooplankton and environmental variables [85]. To reduce the impact of double zeros, we used a lg(x+1)-transformation to the abundance of common zooplankton taxa prior to analysis. Significant environmental variables in the RDA were selected with the Monte Carlo permutation test (999 permutations) [78]. Collinearity between variables was assessed with the variance inflation factor (VIF), which estimates the influence of collinearity among the variables [85].

The relationships between biotic variables and the measured environmental parameters were examined with Statistica version 10.01011.0 software (GLZ—generalized linear models). Being less sensitive regarding the normality and homoscedasticity of data, GLZ is more suitable than GLM. All input variables were lg(x+1)-transformed to fit normality [86]. Therefore, a logarithm link function and normal distribution (f(z) = log(z)) were applied to estimate the effects of all independent variables [87]. The best models were selected based on the Akaike Information Criterion (AIC). To validate the predicted regression models, the Wald statistic was applied to check the significance of r-coefficients for input parameters. A likelihood ratio test was used to assess the statistical significance of including variables. The goodness of fit for each model was evaluated with deviance and Pearson χ2. Relationships between biotic variables were also tested with GLZ.

Contour plots of environmental and biological variables were made using the kriging as a gridding method in Surfer 8.0 (Golden Software Inc., Golden CO, USA).

## 3. Results

### 3.1. Environmental Conditions

#### 3.1.1. Hydrology

**Figure 2:**
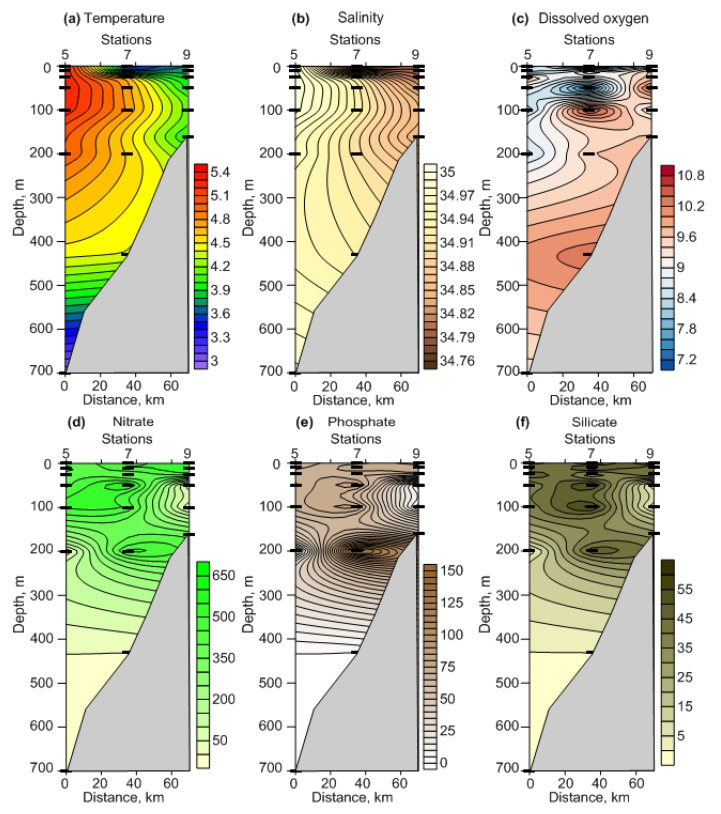
Vertical profiles of water temperature, °C (**a**), salinity, psu (**b**), dissolved oxygen, mL L^−1^ (**c**), nitrate, μg L^−1^ (**d**), phosphate, μg L^−1^ (**e**), and silicate, μg L^−1^ (**f**) in the Fram Strait, winter 2021.

Both regions had no ice cover during the study period. Sampling stations in FS were mainly affected by relatively warm AW (Figure 2a,b). The vertical profiles of temperature and salinity indicated that the upper and intermediate layers were well-mixed, and that the water temperatures above 400 m varied from 3.5 to 5.3 °C (Figure 2a,b).

**Figure 3:**
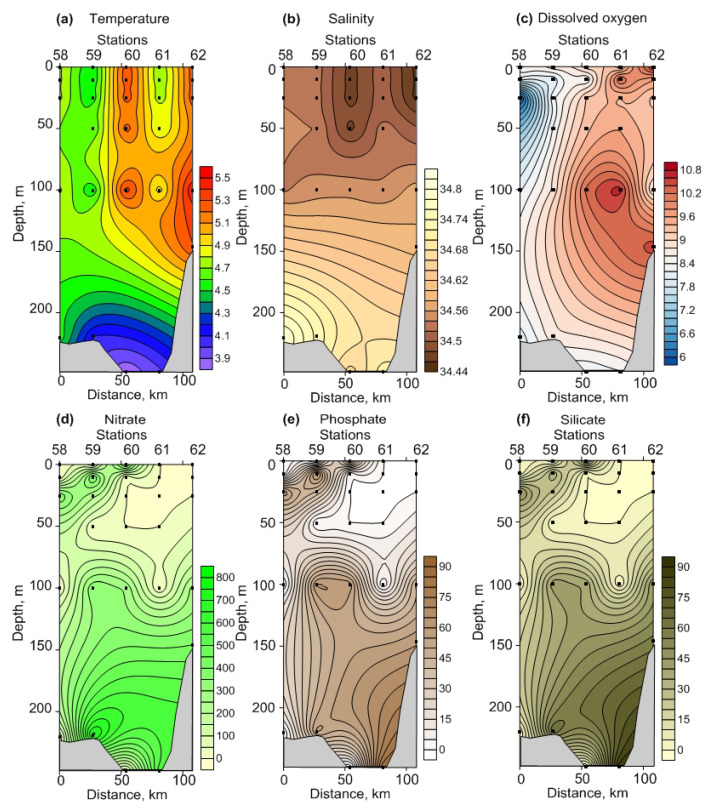
Vertical profiles of water temperature, °C (**a**), salinity, psu (**b**), dissolved oxygen, mL L^−1^ (**c**), nitrate, μg L^−1^ (**d**), phosphate, μg L^−1^ (**e**), and silicate, μg L^−1^ (**f**) in the Southern Barents Sea, winter 2021.

There was a spatial gradient in the surface water temperature; it tended to decrease from south to north (Figure 2a). The salinity demonstrated a slight variation in the whole water column, with lower values in the upper 10 m layer (Figure 2b). In BS, AW was also present at all sampling stations (Figure 3a,b). There was no clear vertical stratification according to the hydrological properties in the water column (Figure 3a,b). The water temperature increased towards the surface, while salinity showed an inverse pattern (Figure 3a,b). There were clear differences in the salinity between regions (ANOVA or Kruskal-Wallis test, *p* = 0.001–0.008) for each sampling layer. The temperature was similar among the regions (ANOVA or Kruskal-Wallis test, *p* = 0.160–0.680).

#### 3.1.2. Dissolved Oxygen and Nutrients

In FS, the dissolved oxygen concentration was high below 100 m, ranging from 9 to 10.8 mL L^−1^ (Figure 2c). The vertical distribution of nutrients at different depths showed more or less comparable contents at all stations, with the greatest values occurring in the upper 200 m layer (Figure 2d). Nitrate, phosphate, and silicate concentrations ranged from 374 to 624 μg L^−1^ (6–10 μM), from 65 to 143 μg L^−1^ (0.7–1.5 μM), and from 28 to 60 μg L^−1^ (0.4–0.8 μM), respectively, in the surface 50 m layer (Figure 2d–f).

In BS, the dissolved oxygen content (5.8–10.7 mL L^−1^) slightly increased from the north to the south, and this pattern was more evident for the upper 50 m (Figure 3c). Nutrients were concentrated mainly below the 100 m layer, except for the two northernmost stations (Figure 3d–f). The nitrate concentration ranged between 217 and 780 μg L^−1^ (3.5–12.6 μM), with peak levels occurring near the bottom (Figure 3d). Phosphates reached their maxima at the southernmost stations (89 μg L^−1^ or 0.9 μM) in the bottom layers (Figure 3e). Silicates showed a similar pattern to other nutrients, being higher in the deepwater layers, where they amounted to 88 μg L^−1^ (1.2 μM) (Figure 3f). Comparisons of the hydrochemical parameters revealed significant differences between regions in the nitrate and phosphate concentrations (0–25 m layer and 0–bottom layer, ANOVA or Kruskal-Wallis test, *p* = 0.010–0.035) and in the silicate concentrations (0 m layer and 0–bottom layer, ANOVA or Kruskal-Wallis test, *p* = 0.012–0.040). Dissolved oxygen concentrations were similar at both study sites (ANOVA or Kruskal-Wallis test, *p* = 0.15–0.95).

PCA revealed a clear separation of stations in FS and in BS based on the environmental variables (Figure S1, Tables S1 and S2). One-way PERMANOVA validated the spatial separations of regions obtained with PCA (F = 10.36, *p* = 0.0007). Two-way PERMANOVA indicated that sampling layer and combination of region–sampling layer had no significant impact on the separation of the stations (F = −0.85–0.26, *p* = 0.36–0.89)

### 3.2. Plankton Distribution

#### 3.2.1. Microbial Parameters

**Figure 4:**
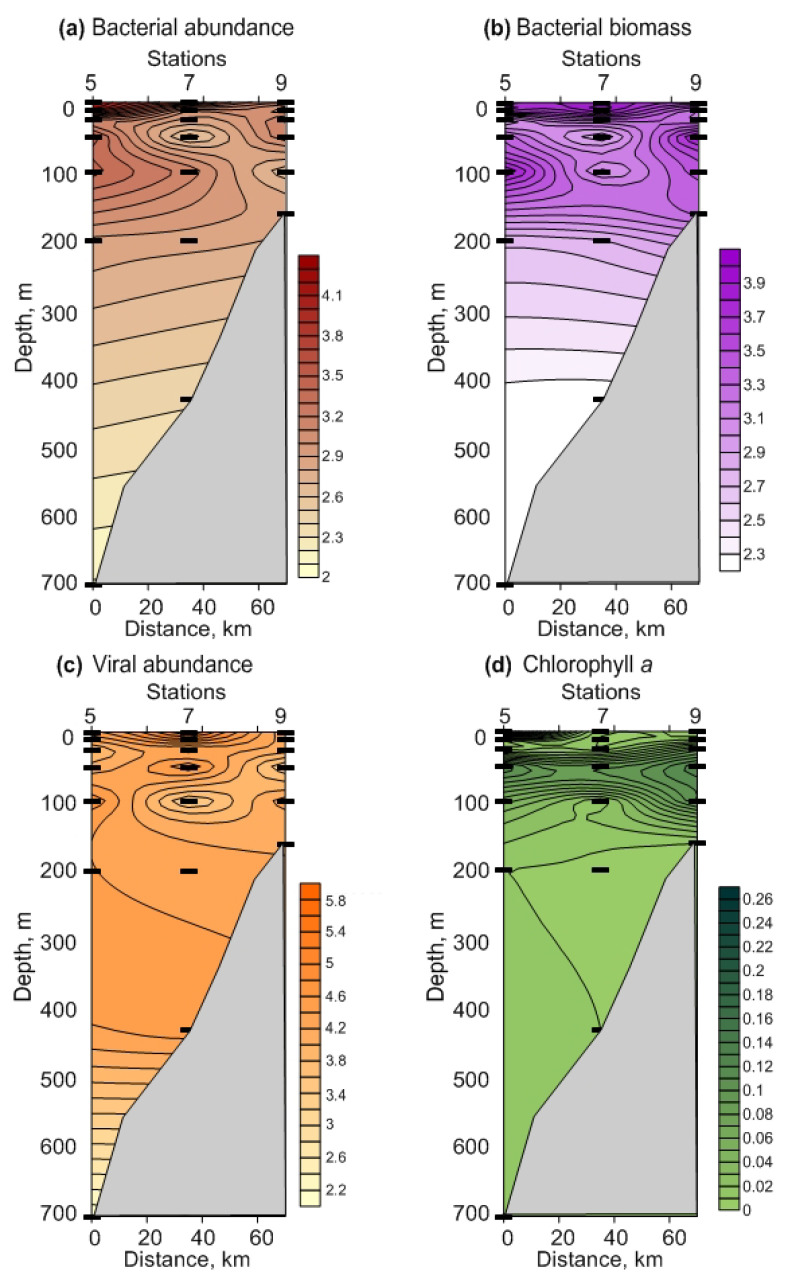
Vertical profiles of bacterial abundance, 10^5^ cells mL^−1^ (**a**), bacterial biomass, mg C m^−3^ (**b**), viral abundance, 106 particles mL^−1^ (**c**), and chlorophyll *a*, mg m^−3^ (**d**) in the Fram Strait, winter 2021.

The abundance of heterotrophic bacteria ranged from 2.1 × 10^5^ to 4.2 × 10^5^ cells mL^−1^ in FS (Table S3, Figure 4a).

**Figure 5:**
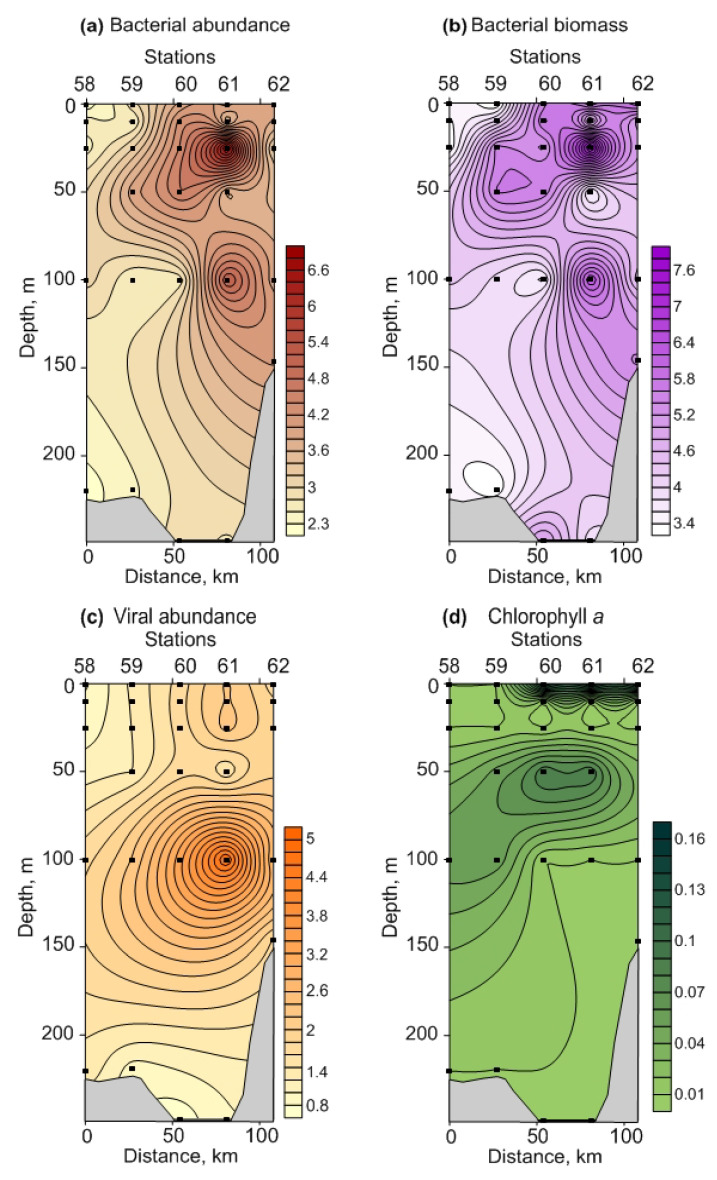
Vertical profiles of bacterial abundance, 10^5^ cells mL^−1^ (**a**), bacterial biomass, mg C m^−3^ (**b**), viral abundance, 10^6^ particles mL^−1^ (**c**), and chlorophyll *a*, mg m^−3^ (**d**) in the Southern Barents Sea, winter 2021.

It slightly decreased in the upper 0–10 m layer northward, while no clear fluctuations were below 100 m (Figure 4a). Bacterial biomass varied from 2.2 to 4.0 mg C m^−3^ (Table S3) and demonstrated the same pattern as the abundance (Figure 4b). In BS, the total bacterial density and biomass were 2.3 × 10^5^–6.8 × 10^5^ cells mL^−1^ and 3.2–7.9 mg C m^−3^, respectively, increasing from the north to the south in the surface and intermediate layers (Figure 5a,b). In both regions, the abundance and biomass of bacteria declined towards the bottom (Figure 4a,b and Figure 5a,b). Significant regional differences in the total bacterial biomass for the bottom and 0–bottom layers were revealed (Table S3). Bacterial abundances were comparable in both regions (Table S3).

The volume of bacterial cells (AVB) varied from 0.026 to 0.049 μm^3^, averaging 0.035 ± 0.006 μm^3^ in FS. It slightly decreased with depth. In BS, AVB was 0.031–0.070 μm^3^, with a mean of 0.05 ± 0.01 μm^3^. Significant regional differences were found according to one-way ANOVA (F = 33.49. *p* = 0.0001).

Vertical profiles of viral abundance are indicated in Figure 4c and Figure 5c. In the surface and intermediate waters of FS, the abundance of virus particles was the highest, ranging from 3.3 · 10^6^ to 5.7 · 10^6^ viruses mL^−1^. The bottom layers displayed lower viral abundances (2.0 × 10^6^–4.5 × 10^6^ viruses mL^−1^) (Figure 4c, Table S3). In BS, most of the viruses were located at the 100 m layer (1.7 × 10^6^–4.9 × 10^6^ viruses mL^−1^) while the surface and bottom abundances were lower (0.7 × 10^6^–2.2 · 10^6^ and 0.7 × 10^6^–1.8 × 10^6^ viruses mL^−1^, respectively) (Figure 5c, Table S3). Coastal stations (st. 61 and 62) showed a greater viral abundance compared to more oceanic stations (Figure 5c). Significant differences in the abundances of viruses were found for all sampling layers (Table S3), except for the 100 m layer (Table S3).

The virus-to-bacteria ratio (VBR) measured from the total bacterial and viral abundances varied significantly (Kruskal-Wallis test, *p* = 0.001) between the study regions, with a greater VBR (10.7–18.7, 13.5 ± 4.6) observed in FS. In BS, VBR fluctuated from 3.0 to 9.2, averaging 4.7 ± 2.2.

#### 3.2.2. Chlorophyll *a*

The Chl-a concentration oscillated weakly between stations in FS (0.01–0.25 mg m^−3^), with higher values in the surface layer (Figure 4d). The bottom estimates were close to 0.01 mg m^−3^. In BS, Chl-a concentrations ranged from 0.01 to 0.15 mg m^−3^, with the maximum measures in the upper and intermediate (50 m) layers (Figure 5d). Chl-a content tended to increase towards the inshore waters (st. 61 and 62) (Figure 5d). The mean values were comparable in both regions (0.05 ± 0.02 mg m^−3^ in FS vs. 0.04 ± 0.02 mg m^−3^ in BS). ANOVA revealed no significant differences between all stations and sampling layers (*p* = 0.11–0.96).

#### 3.2.3. Zooplankton

Pronounced spatial changes were evident for the total zooplankton abundance and biomass in FS, with the highest values occurring in the south. In the 0–50 m layer, both measures varied over a wide range (Table S3), while rather comparable values were recorded for the 50 m–bottom layer (Table S3). In contrast, the zooplankton abundance and biomass tended to decrease from the north to the south in the 50 m–bottom layer, whereas there was no clear spatial pattern of these variables in the upper 50 m layer (Table S3). Significant regional differences were found in the case of the total zooplankton abundance for the 50 m–bottom layer (Table S3). The total zooplankton biomass in the whole water column was significantly higher in BS (Table S3).

**Figure 6:**
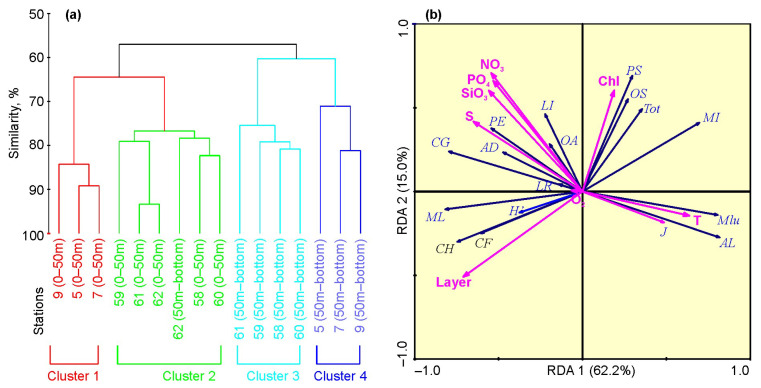
(**a**) Hierarchical dendrogram showing separation of sampling stations based on zooplankton abundance (Bray-Curtis similarity, group-average linkage) in the Fram Strait and in the Southern Barents Sea, winter 2021. (**b**) Results of redundancy analysis (RDA) based on lg(x+1)-transformed zooplankton abundance, and diversity indices in the Fram Strait and in the Southern Barents Sea, winter 2021. Biotic variables: AL—*Acartia longiremis*, CF—*Calanus finmarchicus*, CG—*Calanus glacialis*, CH—*Calanus hyperboreus*, ML—*Metridia longa*, Mlu—*Metridia lucens*, MI—*Microcalanus* spp., OA—*Oithona atlantica*, OS—*Oithona similis*, PS—*Pseudocalanus* spp., AD—*Aglantha digitale*, LI—*Limacina helicina*, LR—*Limacina retroversa*, PE—*Parasagitta elegans*, Tot—Total, J’—Pielou evenness’, H’— Shannon–Wiener diversity. Environmental variables: T—water temperature, S—salinity, Layer—sampling layer, Chl—chlorophyll *a*, O_2_—dissolved oxygen, NO_3_—nitrate, PO_4_—phosphate, SiO_3_—silicate.

A total of 61 zooplankton taxa were identified during the study, with higher species richness in FS relative to BS (54 vs. 39 taxa) (Table 2). Despite strong differences in the number of taxa, diversity estimates were similar in both regions: the Shannon–Wiener index was 3.15 ± 0.07 and 2.95 ± 0.08, and Pielou’s evenness was 0.58 ± 0.01 and 0.61 ± 0.02 in FS and in BS, respectively. Cluster analysis revealed four groups at 57% similarity (Figure 6a, Table 2). These groups showed good correspondence to the regions and sampling layers (Figure 6a, Table 2). Copepods were the only major group in the four clusters constituting >98% of the total zooplankton abundance and >85% of the total biomass. The SIMPER revealed that the dissimilarity between the regions was 33.9% and that the copepods *Acartia longiremis*, *Metridia* spp., *Calanus* spp., *Centropages hamatus, Pseudocalanus* spp., *Microcalanus* spp., *Oithona* spp., *Triconia borealis*, the chaetognath *Parasagitta elegans*, the gastropod *Limacina helicina*, and the appendicularian *Oikopleura* juv. altogether explained >80% of the total variation in the zooplankton communities. Small copepods contributed mostly to the total zooplankton abundance in the upper 50 m layer, while the large-sized taxa occurred in great numbers below 50 m in both regions (Table 2). However, there were clear differences in the contribution of the main taxa, as revealed by the SIMPER analysis. ANOSIM showed significant differences in the zooplankton community structure among the four clusters (Global R = 0.808, *p* = 0.001). Pairwise tests revealed significant differences in zooplankton abundances between regions and layers (R = 0.569–0.741, *p* = 0.008–0.018).

#### 3.2.4. Spatial Separation of the Plankton

PCA indicated that stations sampled in FS and in BS were significantly different based on biotic variables (Figure S2, and Tables S4 and S5). A two-way PERMANOVA based on the biotic variables (bacterial abundance and biomass, Chl-a concentration, and total zooplankton abundance and biomass) indicated significant differences between FS and BS, as well as between sampling layers (F = 5.14–30.47, *p* = 0.0001–0.0004). The post hoc PERMANOVA revealed that most of the pairwise comparisons of the surface and bottom sampling layers were significantly different (*p* = 0.0001–0.0320), whereas the comparisons of the intermediate and upper sampling layers as well as the layers below 200 m were not (*p* = 0.072–0.758).

### 3.3. Environmental Influences on Plankton and Biotic Interactions

Table S6 indicates the results obtained by the generalized linear models (GLZ) from environmental data and biotic variables combined for both regions. Temperature, salinity, and Chl-a content were the factors most influencing spatial variations in the microbial parameters (Table S6). Phytoplankton biomass was strongly associated with hydrological variables, nutrient concentrations, and sampling depth (Table S6). There were some differences between regions in factors explaining variations in microbial parameters (Table S6). In particular, temperature and depth were the most important variables driving spatial changes in the bacterial abundance and biomass, as well as viral abundance in FS, while salinity and phytoplankton biomass explained a significant portion of spatial patterns in microbial variables in BS (Table S6). Nutrients were responsible for changes in Chl-a content in FS, while dissolved oxygen was positively correlated to phytoplankton biomass in BS (Table S6)

The RDA extracted two significant ordination axes, explaining together 77.2% of the total variability of the zooplankton abundance (Figure 6b). The first axis was strongly correlated with hydrological parameters and depth, while the second axis demonstrated a close relation to the nutrient concentrations (Figure 6b).

According to the Monte Carlo permutations test, the variables that had a significant impact on the zooplankton structure were depth and temperature (Table S7). The large copepod species (*Calanus* spp., *Metridia longa*) tended to be positively associated with salinity and depth (Figure 6b). Small copepods (*Oithona* spp. and *Pseudocalanus* spp.) were positively correlated with Chl-a content (Figure 6b). *Acartia longiremis* and *Metridia lucens* demonstrated increasing abundances with temperature (Figure 6b).

Our GLZ models used to assess the biological interactions among plankton groups revealed that bacterial abundance was closely related to Chl-a biomass, and was negatively associated with ABV in both regions (Table S8). The bacterial biomass was positively correlated with ABV. Viral abundance tended to increase with increasing VBR (Table S8). ABV was negatively related to bacterial abundance and Chl-a content, and positively to bacterial biomass (Table S8). Chl-a biomass was associated with most of the biotic variables (Table S8). Fluctuations in zooplankton abundance were best explained via Chl-a biomass (Table S8). In contrast, the total zooplankton biomass was negatively correlated with Chl-a concentration. In addition, it was associated with bacterial abundance and biomass, as well as ABV (Table S8). The responses of the plankton groups differed between regions, with a greater role of Chl-a as a structuring factor of microbial communities and zooplankton in BS (Table S8). In FS, bacterial abundance and biomass were positively correlated with viral abundance, whereas in BS, it had no significant impact on the marine bacteria (Table S8).

## 4. Discussion

### 4.1. Environmental Conditions

Marked climatic fluctuations have been observed in the Arctic in recent years [4,17]. Sea ice cover has dramatically reduced since 1981 in the entire BS [1,28]. The highest decline of sea ice occurred in the Northern BS, and in the eastern Svalbard waters [3]. A stronger warming of the seawater was detected in the regions which are influenced by AW [2,5]. BS has become warmer in the last decade [1,6,12]. The intensity of Atlantic flow into BS and FS is reported to be clearly associated with atmospheric variability over the North Atlantic [2,5,17,88]. Moreover, this trend is proposed to continue into the future [11]. The North Atlantic Oscillation (NAO) is a measure to estimate the atmospheric influence of the climatic conditions in the northern hemisphere [28]. Positive NAO phases are related to the stronger heat transport into BS, increased mean annual water temperature, and the subsequent decline of ice extent [29]. The winter and annual NAO indices demonstrated clear fluctuations during the period of 2000–2021, and the long-term trend was close to zero. The period of anomalously positive events can be noticed in 2014–2020 [5,11]. Moreover, warming processes caused an increase in the total area of open water and led to a prolonged duration of the open water season [8,9,12,17]. Considering a strong impact of water temperature on plankton assemblages, a general warming of the marine environment in the Arctic would have a great influence on the main plankton populations, and we revealed some responses of the plankton to the temperature variations.

Our study covered regions which can be considered as the areas where these warming impacts were the most pronounced. We revealed a similarity in water temperature at both locations. Comparisons of water temperature with previous multi-annual observations [2,37,88] showed greater winter values in both study regions, suggesting a remarkable warming of the whole water column. Moreover, the annual average temperature of AW in the Kola Section (69°30′–77°30′ N, 33°30′ E) was typical of warm years, and exceeded the multi-year mean (1981–2010) by 0.4 °C [5]. In contrast, the salinity in BS and adjacent waters has shown minor changes over the past decade [2,5]. Our study indicated a significantly higher salinity in FS relative to BS. Such differences can partly be explained by a greater freshwater impact from the Kola Peninsula. In particular, the nearshore waters could be affected by less saline waters from Kola Bay toward the open sea. The distribution of water masses in FS and in BS showed a good correspondence to recent observations [2,5,38], with AW occurring in the whole water column.

In the Arctic waters, there are regular seasonal cycles in major nutrients [7,8,9,26,29]. The maximum values of nitrate, phosphate, and silicate concentrations can be detected in the winter season as a result of a reduced photosynthetic activity. A considerable reduction in nutrients occurs in the spring period due to phytoplankton bloom [30,89,90]. During the summer period, phosphate, nitrate, and silicate remain low, and this is associated with seasonal phytoplankton succession in the Arctic marine environment [24,38]. The autumn season is the period when nutrient concentrations tend to increase [26,29,30]. Our study revealed the enhanced contents of nutrients in seawater relative to the summer and autumn observations [29,51,89,90,91,92]. This pattern can be explained by low phytoplankton biomasses recorded in both regions, and the effective regeneration of the nutrients in the water column. Moreover, our measures for nutrients were higher compared to data recorded in FS during the winter of 2014 when nitrate and phosphate accounted for 7.5–10.0 μM and 0.6–0.8 μM, respectively [38]. It is more likely that interannual variations in the intensity of AW influx in the Arctic may be responsible for such differences. Recent observations suggest a stronger inflow of nutrient-rich AW into BS and adjacent waters [5,9]. Vertical distributions of the nutrients in FS in the winter period of 2021 were rather homogenous, indicating a strong impact of vertical mixing. In contrast, in BS, there were maxima of nutrients in the intermediate layers at the nearshore stations. The possible reasons for the observed pattern are the inflow of less saline waters from Kola Bay and adjacent coastal sites in the upper layers, and local circulations in the coastal zone [28,59].

### 4.2. Microbial Parameters

Our study provided novel data regarding the abundance of marine bacteria in two Arctic regions during the period of the polar night. Our estimates in FS (2–4 × 10^5^ cells mL^−1^) were lower than the summer measures (6–17 × 10^5^ cells mL^−1^) reported previously for the West Spitsbergen Current, but comparable with the abundance of 3 × 10^5^ cells mL^−1^ in the East Greenland Current [92]. Other studies have detected that bacterial abundances in AW of FS in summer and autumn were greater compared to our values [93,94,95,96,97,98]. In the Southern BS, summer bacterial abundances fluctuate over a wide range, but they usually exceed 10 × 10^5^ cells mL^−1^ [99]. Our data suggest the winter bacterial abundance and biomass to be lower than in the most productive seasons (spring and summer). However, these (2–4 × 10^5^ cells mL^−1^) were comparable with earlier winter data recorded in the Murmansk coastal waters (1–7 × 10^5^ cells mL^−1^) [26,100]. A comparison of our data with winter estimates from the central Arctic Ocean revealed higher winter abundances and biomasses in FS and in BS (2.5–6.8 × 10^5^ cells mL^−1^ vs. 2.5–6.8 × 10^5^ cells mL^−1^ and 2.8–7.9 mg C m^−3^ vs. 2.6–5.9 mg C m^−3^) [101]. Regional variability in the total bacterial number and biomass is a well-documented phenomenon for the Arctic and other marine environments, and it is thought to be associated with differences in resource availability or predation [33,65,96,102]. In general, we established that the distribution of bacterial abundance and biomass was rather homogenous in the whole water column, with a slight tendency to increase in the upper 50 m layer in both regions. The nearshore waters in BS also demonstrated enhanced bacterial abundance in the intermediate layers. Our study documented a clear regional variability in the ABV, with larger bacterial cells in BS. This discrepancy suggests spatial differences in microbial communities in the two Arctic locations. Recent studies have reported a clear seasonal succession of the microbial composition in FS [65,96,97,98]. It should be noted that the plankton samples in FS and in BS were collected in late November and mid-December, respectively. Therefore, differences in microbial communities between the regions are expected and are in line with the mentioned observations.

The analysis of virioplankton in FS and in BS provided new insights into the current knowledge of the microbial plankton during the period of the polar night. Previous data regarding viral abundance in the Arctic are scarce. In the coastal waters of BS, the winter abundance of virus-like particles was estimated to be 2.5 × 10^6^ viruses mL^−1^ [100], which is comparable with our estimates. At the same time, the concentrations of viruses in the summer and autumn seasons have been reported to be higher, relative to our data [60,99,103]. Stocks of marine viruses were located in the upper 50 m layer in FS, whereas they concentrated in the intermediate 100 m layer in BS. The most prominent difference was the significantly higher viral abundance in FS, where it was 1.4–3.5 times greater. Our study revealed a higher VBR in FS, most likely suggesting lower burst sizes and rates of virally mediated mortality in BS [34,36]. Another possible reason is the dominance of small bacterial cells in FS, providing a greater relative surface for hosting [34,36].

### 4.3. Phytoplankton

Phytoplankton abundance and productivity in BS and other Arctic sites fluctuate considerably throughout the year, with peaks during the spring–summer season [7,8,9,13,30]. Day duration, light regime, nutrient availability, oceanic currents, hydrological factors, grazing impact, and sea ice cover are recognized as the main factors controlling the growth and development of Arctic phytoplankton [7,9,24,29]. The winter season is a less productive period at high latitudes [61,62].

Many previous works have reported extremely low phytoplankton abundance and biomass during the period of the polar night in the BS, FS, and Svalbard waters [11,61,62,63,100,104]. In the present study, we used Chl-a content as a proxy to estimate the total phytoplankton biomass. Our research detected negligible Chl-a concentrations (0.01–0.25 mg m^−3^) in both regions. Comparable Chl-a estimations were obtained recently for FS and Svalbard fjords (<0.025 mg m^−3^) [38,60,63,104] and for the Southern BS (0.03–0.17 mg m^−3^) [100] in the winter periods. Recent studies conducted in more productive seasons (spring and summer) have found generally higher Chl-a biomasses in the Arctic seas [38,46,51]. Our study revealed a stable vertical pattern in the phytoplankton biomass in the water column, with slightly higher concentrations in the upper layers. This was due to the strong vertical mixing that led to homogenous distributions of Chl-a.

### 4.4. Zooplankton

Seasonality in the zooplankton composition in Arctic ecosystems is a well-documented phenomenon connected with environmental fluctuations, food supply, predator pressure, advection, and local circulation [25]. Low zooplankton abundance and biomass have been documented in previous studies during the winter season in the Arctic marine environments [23,45,62,63,104]. Comparisons with other winter studies revealed higher biomass in FS in 2021 relative to the data collected in Svalbard fjords in January 2012 [104]. Our estimates for BS were comparable with the data collected in the same area in November 2012 [23].

Our investigation also indicated low estimates of the total zooplankton abundance and biomass in both regions. The winter zooplankton abundances were 2–15 times and 3–8 times lower than the spring–summer values previously reported in FS and in BS [15,44,49,53,55,105,106]. Despite the dominance of several common taxa, species richness and the diversity of zooplankton assemblages during the period of the polar night may be high and comparable with the estimates in other seasons [21,22,27,37,47,49,50,53,56,57,105]. We also found relatively high species richness, as well as the Shannon-Wiener diversity and the Pielou evenness in both regions. However, the total number of taxa was greater in the FS region. Some possible reasons could explain the higher species richness and zooplankton diversity in the Greenland Sea. First, the plankton fauna of FS included some deepwater species inhabiting waters below 200 m (*Aetideopsis armatus*, *Chiridius obtusifrons*, *Gaetanus tenuispinus*, *Gaidius brevispinus*, *Heterorhabdus norvegicus*, and *Scolecithricella minor*). Second, relative contributions of major taxa were higher in BS, leading to lower H’.

Copepods were found to be the most important members of the zooplankton during the polar night. In our study, they constituted an essential part of the total zooplankton abundance and biomass, showing a good agreement with earlier reports [23,45,62,63,104]. However, there were contrasting patterns in the vertical distributions between taxa with small or large body sizes. The upper 50 m layer was numerically dominated by small- and medium-sized taxa (*Oithona* spp., *Microcalanus* spp., *Microsetella norvegica*, *Pseudocalanus* spp.) while larger species (*Calanus* spp., *Paraeuchaeta* spp., and *Metridia longa*) occurred below 50 m. Differences in the depth distributions can be explained by taking into account the life strategies of the mentioned copepod species. The herbivorous copepods of the genus *Calanus* spp. overwinter at greater depths in diapause during the winter seasons [61,62]. They use lipids to survive severe environmental conditions (mainly the absence of phytoplankton). Omnivorous/detritivores taxa (*Metridia longa*, *Oithona similis*, *Pseudocalanus* spp., and *Microcalanus* spp.) have been found to be active throughout the year [23,45,48,59,63,64,104,107]. Smaller taxa prefer the surface layer, and *Metridia longa* usually occurs in the intermediate and deep water layers [62,64,104,107]. The larger *Paraeuchaeta* spp. are carnivorous copepods which inhabit great depth [37,61,62,108].

Our study detected clear regional differences in the total zooplankton biomass, with greater values in BS. The zooplankton abundance was significantly different only in the 50 m–bottom layer, with lower estimations in FS. It is more likely that bathymetry of the regions could play a role in determining such discrepancies. Deep water regions usually had lower abundances and biomasses compared to the shallower sites, as in the case of the coastal waters in BS.

Recent studies have emphasized the Atlantification of the Arctic regions as the process altering the structures of pelagic communities [1,6]. A poleward shift in the range of marine taxa has been proposed, and some boreal taxa could be found in more northern regions. Our study revealed a rather high abundance of the copepod *Metridia lucens* in FS and in BS, suggesting a more intensive advective impact of AW during recent years. Proportions of other Atlantic taxa were also slightly higher than previously reported [23,45,104]. These findings confirm general observations regarding the borealization process in Arctic pelagic ecosystems.

### 4.5. Environmental Control of Plankton and Biotic Interactions

Our study revealed that hydrological factors and sampling depth have a measurable impact on the plankton groups. However, the importance of each factor was different at a regional scale. The bacterial abundance and biomass were found to covary with water temperature, salinity, and sampling layer, but this influence was stronger in FS. All these factors had a negative influence on the microbial abundance, and the bacterial number tended to decrease with depth and with increasing temperature and salinity. The effect of depth on bacterial abundance has been reported in FS, where rich bacterial assemblages were found in the upper layers [92].

Temperature has been reported as one of the main drivers determining bacterial growth, production, biomass, and abundance in the Arctic seas [96,102]. In our study, the salinity had a greater effect than that of temperature. This may be connected with the low temperature variations in both regions and wider fluctuations in salinity in BS, the region where the lower bacterial abundance and biomass were recorded. Marine bacteria during the polar night were concentrated mainly in the upper 50 m layer where there were more favorable conditions for their growth and development. An inverse relationship between the abundance of bacterioplankton and depth supports our conclusion. Moreover, colder temperatures might have an indirect impact on the bacterial parameters through the availability of resources for heterotrophic prokaryotes [33,101,102]. Lower temperatures may enhance potential sources for bacterioplankton growth, leading to a higher total bacterial abundance [109,110].

The influence of Chl-a on the bacterial abundance was significant in our study, although a strong positive impact on the bacterial biomass was found only for BS. There are some previous evidences that phytoplankton biomass may explain a substantial part of regional variations in bacterial density and production [60,93,94,101]. In contrast, nutrients had no significant influence on bacterial abundance and biomass. It is more likely that the significance of nutrients is higher in the productive seasons, while in the winter, these had lesser impacts.

The size of bacterial cells was one of the main variables explaining the spatial pattern in the total abundance and biomass, while the number of bacteria was correlated negatively with ABV, and the bacterial biomass increased with ABV. This is an expected result because the small-sized bacteria form a more numerous population compared to larger bacteria. In FS, the bacterial abundance and biomass were positively associated with the density of the virioplankton while there were no direct relationships between these variables in BS. Given the prevalence of small bacterial cells in FS, we may propose higher host availabilities for viruses that could cause a rise in their numbers relative to BS where larger bacteria dominated.

ABV was negatively correlated with salinity and temperature, and the larger bacterial cells were concentrated in the intermediate and bottom layers. Similar results have been noted in the Northwestern and Central BS during the autumn–winter periods [60,100]. Three reasons can be proposed to explain this pattern. First, colder temperatures might be favorable for larger bacterial populations. Second, the upper and bottom layers were different in the compositions of bacterial assemblages, with the dominance of smaller groups occurring in the surface layer. Third, large bacteria might sink from the surface to the bottom layers, forming aggregations near the seafloor. Negative correlations between AVB and Chl-a concentrations give additional evidence regarding the higher occurrence of small-sized bacteria in the upper layers where phytoplankton biomass reached maximum values.

Viral abundance and VBR were significantly related to each other and to the biomass of microalgae being greater in the layers, with relatively high Chl-a concentrations. In FS, depth was the most explanatory variable controlling vertical distributions of the virioplankton counts. The mechanism explaining the link between viral abundance, VBR, and Chl-a is not clear. Some studies report no direct correlations between these parameters, while other works provide significant correlations [35,36,60,103]. We may speculate that the higher Chl-a measures reflected enhanced concentrations of microalgae and cyanobacteria that could be favorable for viruses, leading to a higher viral abundance and VBR in the chlorophyll-rich layers.

We found that phytoplankton biomass reckoned as the Chl-a content tended to be increased with temperature, salinity, dissolved oxygen, and nitrate concentrations. Oceanographic conditions and nutrients are among the factors having a significant influence on the growth and development of marine phytoplankton [34,89,90,91]. The effects of temperature and salinity on microalgae have been studied in the spring and summer periods [29,46,51]. These factors are thought to indirectly affect the phytoplankton in the Arctic, causing the stabilization of the water column [7,8,9,11,13]. In the productive seasons, the light regime plays an important role. Temperature probably would have a greater role in the period of darkness, determining the rates of growth, respiration, and other physiological processes. However, special studies focused on the biochemistry and physiology of the Arctic phytoplankton are needed to support our statement. We also revealed an inverse relationship between depth and Chl-a, suggesting that the phytoplankton was located in the upper layers. This result is in accordance with other studies dealing with Arctic microalgae [11,26,30], and suggests that the upper layers are more suitable for phytoplankton growth.

Zooplankton abundance and biomass were positively associated with Chl-a, bacterial abundance, and the size of bacteria in our study. In BS, these were also positively correlated with viral abundance. Food availability can be considered as an important factor affecting zooplankton growth and survival during periods when potential food items are scarce, because environmental variables may have a greater significance under food-saturated conditions [24,25,107]. Therefore, the positive relationship with phytoplankton biomass during the polar night is an expected finding, due to the extremely low Chl-a concentrations. Bacterial aggregations can be used as food resources by many small zooplankton taxa [111]. Small copepods were found to be the most numerous group in our study. They concentrated in the upper layers, with high bacterial abundances. It is more likely that bacterial cells may be utilized by these copepods supporting the direct correlation between zooplankton and bacterial densities [60].

The present study showed that the spatial variation in the zooplankton community was controlled by a set of environmental variables, with temperature and depth being the most significant. Common species demonstrated different responses to environmental factors. For instance, small copepods were positively related to phytoplankton biomass and tended to be located at the surface, while the larger species showed an opposite pattern. Coastal species, *Acartia longiremis* and the Atlantic copepod *Metridia lucens,* were strongly correlated with temperature, while *Calanus finmarchicus* was positively related to salinity, indicating a strong association of these taxa with warm AW. Temperature, being an indicator of intensity of AW inflow into the Arctic, is an important factor determining the abundance and biomass of zooplankton taxa throughout the year, as has been revealed in many previous reports [14,15,33,37,55,56,57,58,59,62,106].

Finally, it should be noted that in the winter periods, other factors could have a significant influence on the plankton. In particular, dissolved organic matter provides an important source for microbial assemblages in any aquatic environment [29,102]. Bacterivorous heterotrophic eukaryotes (may control the abundances and biomass of marine prokaryotes through grazing [102], and this impact may be significant in the polar night. On the other hand, heterotrophic protists can be an important food source for larger zooplankton (copepods, appendicularians, and *Limacina* spp.) supporting their populations during the winter season [63,104,111].

## 5. Conclusions

The Arctic marine environments have been strongly changed over the past few decades due to strong climatic perturbations. Pelagic communities are good indicators of environmental forcing, and they may quickly respond to variations in natural conditions. The polar night is a unique phenomenon in the Arctic and Antarctic. Despite recent advances in marine ecology, many aspects regarding the structure and functioning of plankton assemblages during the period of the polar night remain unexplored. Our study presents novel data on the distribution of important plankton groups in the Fram Strait and in the Barents Sea, two major pathways connecting the Atlantic and Arctic oceans. We initially hypothesized that both regions should be similar in environmental and plankton characteristics. However, despite the similarities in temperature, bacterial abundance, and chlorophyll *a*, the two localities were significantly different, based on the whole dataset. Vertical patterns of the total bacterial abundance and biomass demonstrated a homogenous distribution in the water column, although the upper layers were colonized with more abundant populations. The mean size of bacterial cells was significantly higher in the Southern Barents Sea, suggesting potential regional differences in the composition of marine bacteria. Viral abundance and the virus-to-bacteria ratios tended to be higher in the Fram Strait. Phytoplankton biomass was found to be extremely low during the study period, but it had a considerable impact on the plankton. Zooplankton assemblages were dominated by copepods, demonstrating contrasting distributions in relation to individual body size, so that small taxa were common inhabitants in the upper layers, while larger taxa occurred mainly below 50 m. Temperature, salinity, and the depth of sampling were the most important drivers determining spatial fluctuations in the plankton variables, while nutrients played a minor role. Biotic parameters were correlated with each other, demonstrating strong links between all components of the plankton, from viruses to copepods. Our study expands the current knowledge regarding the structure and ecology of Arctic marine plankton during the period of the polar night, and it may be used as a baseline for future research dealing with the functioning of pelagic food webs.

## Figures and Tables

**Table 1:** List of sampling stations in the Fram Strait and in the Southern Barents Sea, winter 2021.

Station	Date	Region	N	E	Depth, m (Number of Sampling Layers)	Number of Samples (Viruses/ Bacteria/ Chl *a*/ Zooplankton)
5	24 November 2021	Fram Strait	78°40′	08°30′	700 (7)	5/7/6/2
7	24 November 2021	Fram Strait	79°00′	08°30′	430 (7)	5/7/6/2
9	25 November 2021	Fram Strait	79°20′	08°30′	161 (6)	5/6/8/2
58	16 December 2021	Southern Barents Sea	70°60′	33°31′	220 (6)	3/5/5/2
59	16 December 2021	Southern Barents Sea	70°45′	33°30′	219 (6)	4/6/5/2
60	17 December 2021	Southern Barents Sea	70°30′	33°30′	248 (5)	3/6/5/2
61	17 December 2021	Southern Barents Sea	70°15′	33°30′	248 (5)	5/6/6/2
62	18 December 2021	Southern Barents Sea	70°00′	33°29′	146 (5)	3/5/5/2

**Table 2:** Zooplankton abundance and biomass in the Fram Strait and in the Southern Barents Sea, winter 2021.

	Fram Strait		Barents Sea	
	Abundance	Biomass	Abundance	Biomass
Taxon	0–50 m/ 50 m–bottom	0–50 m/ 50 m–bottom	0–50 m/ 50 m–bottom	0–50 m/ 50 m–bottom
**Copepoda**				
*Acartia longiremis*	2 ± 0/<1	<0.1/<0.1	44 ± 18/7 ± 3	0.4 ± 0.2/0.1 ± 0
*Aetideopsis armatus*	-/<1	-/<0.1	-/-	-/-
*Bradyidius similis*	<1/<1	<0.1/<0.1	-/<1	-/<0.1
*Calanus finmarchicus*	20 ± 7/22 ± 10	3 ± 1/3 ± 1	8 ± 4/162 ± 42	1.3 ± 0.8/34 ± 9.2
*Calanus glacialis*	38 ± 21/14 ± 4	6.8 ± 4/3.7 ± 1.5	1 ± 1/17 ± 4	0.2 ± 0.1/3.6 ± 1.1
*Calanus hyperboreus*	1 ± 0/3 ± 1	0.1 ± 0/0.9 ± 0.5	<1/2 ± 1	<1/0.3 ± 0.1
*Centropages hamatus*	-/-	-/-	2 ± 1/1 ± 0	<0.1/<0.1
*Chiridius obtusifrons*	-/<1	-/<0.1	-/-	-/-
Copepoda nauplii	<1/<1	<0.1/<0.1	-/-	-/-
*Gaetanus tenuispinus*	-/1 ± 0	-/0.2 ± 0.1	-/-	-/-
*Gaidius brevispinus*	-/<1	-/<0.1	-/-	-/-
*Heterorhabdus norvegicus*	-/<1	-/<0.1	-/-	-/-
*Jaschnovia brevis*	-/<1	-/<0.1	-/-	-/-
*Metridia longa*	13 ± 3/11 ± 3	1.4 ± 0.4/1.1 ± 0.2	1 ± 0/46 ± 18	0.1 ± 0.1/4.5 ± 1.9
*Metridia lucens*	14 ± 3/4 ± 1	0.5 ± 0.2/0.1 ± 0	129 ± 48/27 ± 11	3.5 ± 1.4/0.7 ± 0.4
*Microcalanus pusillus*	32 ± 7/7 ± 4	<0.1/<0.1	76 ± 10/32 ± 16	0.1 ± 0/<0.1
*Microcalanus pygmaeus*	88 ± 8/12 ± 2	0.1 ± 0/<0.1	51 ± 8/35 ± 7	0.1 ± 0/<0.1
*Microsetella norvegica*	-/-	-/-	<1/-	<0.1/-
*Oithona atlantica*	10 ± 3/7 ± 4	<0.1/<0.1	3 ± 1/10 ± 4	<0.1/<0.1
*Oithona similis*	364 ± 66/67 ± 33	0.4 ± 0.1/0.1 ± 0	177 ± 25/122 ± 34	0.2 ± 0/0.1 ± 0
*Triconia borealis*	1 ± 1/1 ± 0	<0.1/<0.1	-/-	-/-
*Paraeuchaeta* spp. I–IV	-/1 ± 0	-/0.2 ± 0.1	-/<1	-/<0.1
*Paraeuhaeta glacialis* V–VI	<1/<1	<0.1/0.7 ± 0.2	-/<1	-/<0.1
*Paraeuhaeta norvegica* V–VI	<1/1 ± 0	<0.1/0.8 ± 0.5	-/<1	-/<0.1
*Pseudocalanus* spp. I–IV	175 ± 33/33 ± 4	0.6 ± 0.1/0.1 ± 0	87 ± 8/14 ± 6	0.3 ± 0/0.1 ± 0
*Pseudocalanus minutus* V–VI	108 ± 23/31 ± 3	1 ± 0.2/0.3 ± 0	12 ± 2/14 ± 5	0.1 ± 0/0.1 ± 0.1
*Pseudocalanus acuspes* V–VI	41 ± 7/14 ± 3	0.3 ± 0.1/0.1 ± 0	8 ± 2/9 ± 6	0.1 ± 0/0.1 ± 0
*Scolecithricella minor*	-/1 ± 0	-/<0.1	-/-	-/-
*Temora longicornis*	-/-	-/-	<1/<1	<0.1/<0.1
**Ostracoda**				
*Boroecia borealis*	<1/1 ± 0	<0.1/<0.1	-/-	-/-
*Discoconchoecia elegans*	-/<1	-/<0.1	-/-	-/-
*Boroecia maxima*	-/<1	-/<0.1	-/-	-/-
**Amphipoda**				
*Themisto abyssorum*	-/-	-/-	-/<1	-/<0.1
*Themisto libellula*	-/<1	-/0.1 ± 0.1	-/-	-/-
**Euphausiacea**				
*Meganyctyphanes norvegica*	-/<1	-/0.4 ± 0.1	-/<1	-/0.2 ± 0.1
*Thysanoessa inermis*	-/<1	-/0.6 ± 0.2	-/<1	-/0.1 ± 0
*Thysanoessa longicaudata*	<1/<1	<0.1/0.2 ± 0.1	<1/<1	<0.1/<0.1
*Nematoscelis megalops*	-/<1	-/<0.1	-/-	-/-
*Themisto abyssorum*	-/-	-/-	-/<1	-/<0.1
*Themisto libellula*	-/<1	-/0.1 ± 0.1	-/-	-/-
**Decapoda**				
*Pandalus borealis* larvae	-/<1	-/<0.1	-/-	-/-
**Cnidaria**				
*Aglantha digitale*	<1/1 ± 0	<0.1/0.2 ± 0	<1/<1	<0.1/0.2 ± 0
*Dimophyes arctica*	<1/<1	<0.1/<0.1	-/-	-/-
*Rathkea octopunctata*	<1/-	<0.1/-	-/-	-/-
*Physophora hydrostatica* necthophore	-/<1	-/<0.1	<1/<1	<0.1/<0.1
*Pandalus borealis* larvae	-/<1	-/<0.1	-/-	-/-
**Ctenophora**				
*Beroe cucumis*	-/-	-/-	<1/<1	<0.1/<0.1
*Mertensia ovum*	<1/<1	<0.1/0.1 ± 0.1	<1/<1	<0.1/<0.1
*Pleurobrachia pileus*	-/<1	-/<0.1	-/-	-/-
**Polychaeta**				
*Pelagobia longicirrata*	<1/<1	<0.1/<0.1	-/<1	-/<0.1
*Tomopteris helgolandica*	-/<1	-/<0.1	-/<1	-/<0.1
Polychaeta larvae	1 ± 0/-	<0.1/-	-/<1	-/<0.1
**Nemertini**				
Nemertini juv.	-/-	-/-	-/<1	-/<0.1
**Gastropoda**				
*Clione limacina* larvae	<1/<1	<0.1/<0.1	-/-	-/-
*Clione limacina*	<1/<1	<0.1/0.3 ± 0.1	-/-	-/-
*Limacina helicina* larvae	4 ± 1/2 ± 1	0.2 ± 0.1/0.1 ± 0.1	1 ± 0/1 ± 0	<0.1/0.1 ± 0
*Limacina helicina*	1 ± 0/1 ± 0	0.1 ± 0.1/0.1 ± 0	<1/1 ± 0	<0.1/0.2 ± 0
*Limacina retroversa*	1 ± 0/<1	0.1 ± 0/<0.1	<1/1 ± 0	<0.1/0.1 ± 0
Gastropoda larvae	-/<1	-/<0.1	<1/<1	<0.1/<0.1
**Chaetognatha**				
*Eukrohnia hamata*	-/<1	-/0.4 ± 0.2	-/<1	-/0.3 ± 0.2
*Parasagitta elegans*	2 ± 0/3 ± 2	1.3 ± 0.3/1.6 ± 1	<1/<1	<0.1/0.8 ± 0.3
**Appendicularia**				
*Oikopleura* juv.	2 ± 0/<1	<0.1/<0.1	<1/-	<0.1/-
*Oikopleura vanhoeffeni*	<1/<1	<0.1/<0.1	-/-	-/-

Note. ‘-’—taxon not found.

## Data Availability

The data are available on request from the corresponding author.

## Supplementary Materials

The following are available online at: https://www.mdpi.com/article/10.3390/biology12030368/s1, Figure S1: Results of principal component analysis (PCA): ordination diagram indicating separations of sampling stations based on hydrological variables (water temperature and salinity) and hydrochemical parameters (dissolved oxygen, nitrate, phosphate, and silicate) in the Barents Sea and the Fram Strait, winter 2021. Table S1: Factor scores extracted with principal component analysis (PCA) based on hydrological variables (water temperature and salinity) and hydrochemical parameters (dissolved oxygen, nitrate, phosphate, and silicate) in the Barents Sea and the Fram Strait, winter 2021. Table S2: Factor loadings extracted with principal component analysis (PCA) based on hydrological variables (water temperature and salinity) and hydrochemical parameters (dissolved oxygen, nitrate, phosphate, and silicate) in the Barents Sea and the Fram Strait, winter 2021. Table S3: Plankton abundance and biomass (range and mean ± SE) in the Fram Strait and in the Southern Barents Sea, winter 2021. Comparisons were performed using one-way ANOVA or Kruskal-Wallis test, significant differences at *p* < 0.05. n/a—no analysis. Figure S2: Results of principal component analysis (PCA): ordination diagram indicating separations of sampling stations based on biotic variables (bacterial abundance and biomass, viral abundance, chlorophyll *a* concentration, and zooplankton abundance and biomass) in the Barents Sea and the Fram Strait, winter 2021. Table S4: Factor scores extracted with principal component analysis (PCA) based on biotic variables (bacterial abundance and biomass, viral abundance, chlorophyll *a* concentration, and zooplankton abundance and biomass) in the Barents Sea and the Fram Strait, winter 2021. Table S5: Factor loadings extracted with principal component analysis (PCA) based on biotic variables (bacterial abundance and biomass, viral abundance, chlorophyll *a* concentration, and zooplankton abundance and biomass) in the Barents Sea and the Fram Strait, winter 2021. Table S6: Results of 18 GLZ models with microplankton characteristics and chlorophyll *a* concentration as the dependent variables, and environmental parameters as independent variables in the Fram Strait and in the Southern Barents Sea, winter 2021. Only significant explaining variables (*p* < 0.05) are indicated. Table S7: Ranking of environmental variables influenced zooplankton assemblages in the Fram Strait and in the Southern Barents Sea, winter 2021 (Monte Carlo permutation test, 999 permutations). Significant differences was set at *p* < 0.05. Table S8: Results of 24 GLZ models showing interrelations between biotic variables in the Fram Strait and in the Southern Barents Sea, winter 2021. Only significant explaining variables (*p* < 0.05) are indicated.

## References

[1] Polyakov I.V., Pnyushkov A., Alkire M., Ashik I.M., Baumann T.M., Carmack E.C., Goszczko I., Guthrie J.D., Ivanov V.V., Kanzow T. (2017). Greater role for Atlantic inflows on sea-ice loss in the Eurasian Basin of the Arctic Ocean. Science.

[2] ICES (2021). Working Group on the Integrated Assessments of the Barents Sea (WGIBAR). ICES Sci. Rep..

[3] Isaksen K., Nordli Ø., Ivanov B., Køltzow M.A., Aaboe S., Gjelten H.M., Mezghani A., Eastwoodl S., Førland E., Benestad R.E. (2022). Exceptional warming over the Barents area. Sci. Rep..

[4] Rantanen M., Karpechko A.Y., Lipponen A., Nordling K., Hyvärinen O., Ruosteenoja K., Vihma T., Laaksonen A. (2022). The Arctic has warmed nearly four times faster than the globe since 1979. Commun. Earth Environ..

[5] ICES (2022). Working Group on the Integrated Assessments of the Barents Sea (WGIBAR). ICES Sci. Rep..

[6] Polyakov I.V., Alkire M.B., Bluhm B.A., Brown K.A., Carmack E.C., Chierici M., Danielson S.L., Ellingsen I., Ershova E.A., Gårdfeldt K. (2020). Borealization of the Arctic Ocean in response to anomalous advection from sub-arctic seas. Front. Mar. Sci..

[7] Arrigo K.R., van Dijken G.L. (2015). Continued increases in Arctic Ocean primary production. Prog. Oceanogr..

[8] Lewis K.M., Van Dijken G.L., Arrigo K.R. (2020). Changes in phytoplankton concentration now drive increased Arctic Ocean primary production. Science.

[9] Dalpadado P., Arrigo K.R., van Dijken G.L., Skjoldal H.R., Bagøien E., Dolgov A.V., Prokopchuk I.P., Sperfeld E. (2020). Climate effects on temporal and spatial dynamics of phytoplankton and zooplankton in the Barents Sea. Progr. Oceanogr..

[10] Slagstad D., Wassmann P., Ellingsen I. (2015). Physical constrains and productivity in the future Arctic Ocean. Front. Mar. Sci..

[11] Dvoretsky V.G., Vodopianova V.V., Bulavina A.S. (2023). Effects of Climate Change on Chlorophyll *a* in the Barents Sea: A Long-Term Assessment. Biology.

[12] Lind S., Ingvaldsen R.B., Furevik T. (2018). Arctic warming hotspot in the northern Barents Sea linked to declining sea-ice import. Nat. Clim. Chang..

[13] Ardyna M., Arrigo K. (2020). Phytoplankton dynamics in a changing Arctic Ocean. Nat. Clim. Change.

[14] Dvoretsky V.G., Dvoretsky A.G. (2023). Copepod assemblages in a large Arctic coastal area: A baseline summer study. Diversity.

[15] Dvoretsky V.G., Dvoretsky A.G. (2013). Epiplankton in the Barents Sea: Summer variations of mesozooplankton biomass, community structure and diversity. Cont. Shelf Res..

[16] Ingvaldsen R.B., Assmann K.M., Primicerio R., Fossheim M., Polyakov I.V., Dolgov A.V. (2021). Physical manifestations and ecological implications of Arctic Atlantification. Nat. Rev. Earth Environ..

[17] Tesi T., Muschitiello F., Mollenhauer G., Miserocchi S., Langone L., Ceccarelli C., Capotondi L. (2021). Rapid Atlantification along the Fram Strait at the beginning of the 20th century. Sci. Adv..

[18] Evseeva O.Y., Ishkulova T.G., Dvoretsky A.G. (2022). Environmental drivers of an intertidal bryozoan community in the Barents Sea: A case study. Animals.

[19] Evseeva O.Y., Dvoretsky A.G. (2023). Shallow-water bryozoan communities in a glacier fjord of West Svalbard, Norway: Species composition and effects of environmental factors. Biology.

[20] Eriksen E., Skjoldal H.R., Gjøsæter H., Primicerio R. (2017). Spatial and temporal changes in the Barents Sea pelagic compartment during the recent warming. Prog. Oceanogr..

[21] Weydmann A., Walczowski W., Carstensen J., Kwaśniewski S. (2018). Warming of Subarctic waters accelerates development of a key marine zooplankton *Calanus Finmarchicus*. Glob. Chang. Biol..

[22] Dvoretsky V.G., Dvoretsky A.G. (2022). Coastal mesozooplankton assemblages during spring bloom in the eastern Barents Sea. Biology.

[23] Dvoretsky V.G., Dvoretsky A.G. (2018). Mesozooplankton in the Kola Transect (Barents Sea): Autumn and winter structure. J. Sea Res..

[24] Raymont J.E.G. (1983). Plankton and Productivity in the Oceans.

[25] Castellani C., Edwards M. (2017). Marine Plankton: A Practical Guide to Ecology, Methodology, and Taxonomy.

[26] Matishov G.G. (1985). Life and Environmental Conditions in the Pelagial of the Barents Sea.

[27] Wassmann P., Reigstad M., Haug T., Rudels B., Carroll M.L., Hop H., Gabrielsen G.W., Falk-Petersen S., Denisenko S.G., Arashkevich E. (2006). Food webs and carbon flux in the Barents Sea. Progr. Oceanogr..

[28] Jakobsen T., Ozhigin V.K. (2011). The Barents Sea: Ecosystem, Resources, Management: Half a Century of Russian-Norwegian Cooperation.

[29] Sakshaug E., Johnsen G., Kovacs K. (2009). Ecosystem Barents Sea.

[30] Makarevich P., Druzhkova E., Larionov V., Mahamane A. (2012). Primary producers of the Barents Sea. Diversity of Ecosystems.

[31] Pavlova L.V., Zuyev Y.A., Dvoretsky A.G. (2023). Shallow-water benthic communities on soft bottoms of a sub-arctic fjord (Southern Barents Sea, Russia) along a gradient of ecological factors. Diversity.

[32] Pavlova L.V., Dvoretsky G. (2022). Prey selectivity in juvenile red king crabs from the coastal Barents Sea. Diversity.

[33] Azam F., Malfatti F. (2007). Microbial structuring of marine ecosystems. Nat. Rev. Microbiol..

[34] Fuhrman J.A., Cram J.A., Needham D.M. (2015). Marine microbial community dynamics and their ecological interpretation. Nat. Rev. Microbiol..

[35] Wommack K.E., Colwell R.R. (2000). Virioplankton: Viruses in aquatic ecosystems. Microbiol. Mol. Biol. Rev..

[36] Zimmerman A.E., Howard-Varona C., Needham D.M., John S.G., Worden A.Z., Sullivan M.B., Waldbauer J.R., Coleman M.L. (2020). Metabolic and biogeochemical consequences of viral infection in aquatic ecosystems. Nat. Rev. Microbiol..

[37] Hop H., Falk-Petersen S., Svendsen H., Kwasniewski S., Pavlov V., Pavlova O., Soreide J.E. (2006). Physical and biological characteristics of the pelagic system across Fram Strait to Kongsfjorden. Prog. Oceanogr..

[38] Randelhoff A., Reigstad M., Chierici M., Sundfjord A., Ivanov V., Cape M., Vernet M., Tremblay J.É., Bratbak G., Kristiansen S. (2018). Seasonality of the Physical and Biogeochemical Hydrography in the Inflow to the Arctic Ocean through Fram Strait. Front. Mar. Sci..

[39] Dvoretsky A.G., Dvoretsky V.G. (2016). Inter-annual dynamics of the Barents Sea red king crab (*Paralithodes camtschaticus*) stock indices in relation to environmental factors. Polar Sci..

[40] Dvoretsky A.G., Dvoretsky V.G. (2022). Epibiotic communities of common crab species in the coastal Barents Sea: Biodiversity and infestation patterns. Diversity.

[41] Dvoretsky A.G., Dvoretsky V.G. (2023). Epibionts of an introduced king crab in the Barents Sea: A second five-year study. Diversity.

[42] Dvoretsky A.G., Dvoretsky V.G. (2015). Commercial fish and shellfish in the Barents Sea: Have introduced crab species affected the population trajectories of commercial fish?. Rev. Fish Biol. Fish..

[43] Dvoretsky A.G., Dvoretsky V.G. (2020). Effects of environmental factors on the abundance, biomass, and individual weight of juvenile red king crabs in the Barents Sea. Front. Mar. Sci..

[44] Dvoretsky V.G., Dvoretsky A.G. (2013). Structure of mesozooplankton community in the Barents Sea and adjacent waters in August 2009. J. Nat. Hist..

[45] Dvoretsky V.G., Dvoretsky A.G. (2020). Arctic marine mesozooplankton at the beginning of the polar night: A case study for southern and south-western Svalbard waters. Polar Biol..

[46] Nöthig E.M., Ramondenc S., Haas A., Hehemann L., Walter A., Bracher A., Lalande C., Metfies K., Peeken I., Bauerfeind E. (2020). Summertime Chlorophyll *a* and Particulate Organic Carbon Standing Stocks in Surface Waters of the Fram Strait and the Arctic Ocean (1991–2015). Front. Mar. Sci..

[47] Dvoretsky V.G., Dvoretsky A.G. (2010). Checklist of fauna found in zooplankton samples from the Barents Sea. Polar Biol..

[48] Dvoretsky V.G., Dvoretsky A.G. (2009). Spatial variations in reproductive characteristics of the small copepod *Oithona similis* in the Barents Sea. Mar. Ecol. Prog. Ser..

[49] Gluchowska M., Dalpadado P., Beszczynska-Möller A., Olszewska A., Ingvaldsen R.B., Kwasniewski S. (2017). Interannual zooplankton variability in the main pathways of the Atlantic water flow into the Arctic Ocean (Fram Strait and Barents Sea branches). ICES J. Mar. Sci..

[50] Dvoretsky V.G., Dvoretsky A.G. (2015). Early winter mesozooplankton of the coastal south-eastern Barents Sea. Estuar. Coast. Shelf Sci..

[51] Nöthig E.M., Bracher A., Engel A., Metfies K., Niehoff B., Peeken I., Bauerfeind E., Cherkasheva A., Gabler-Schwarz S., Hardge K. (2015). Summertime plankton ecology in Fram Strait a compilation of long- and short-term observations. Polar Res..

[52] Dvoretsky V.G., Dvoretsky A.G. (2022). Ecology and distribution of red king crab larvae in the Barents Sea: A review. Water.

[53] Dvoretsky V.G., Dvoretsky A.G. (2012). Estimated copepod production rate and structure of mesozooplankton communities in the coastal Barents Sea during summer–autumn 2007. Polar Biol..

[54] Trudnowska E., Sagan S., Błachowiak-Samołyk K. (2018). Spatial variability and size structure of particles and plankton in the Fram Strait. Prog. Oceanogr..

[55] Dvoretsky V.G., Dvoretsky A.G. (2010). Mesozooplankton structure in Dolgaya Bay (Barents Sea). Polar Biol..

[56] Dvoretsky V.G., Dvoretsky A.G. (2009). Summer mesozooplankton distribution near Novaya Zemlya (eastern Barents Sea). Polar Biol..

[57] Dvoretsky V.G., Dvoretsky A.G. (2009). Summer mesozooplankton structure in the Pechora Sea (south-eastern Barents Sea). Estuar. Coast. Shelf Sci..

[58] Dvoretsky V.G., Dvoretsky A.G. (2022). Summer-fall macrozooplankton assemblages in a large Arctic estuarine zone (south-eastern Barents Sea): Environmental drivers of spatial distribution. Mar. Environ. Res..

[59] Dvoretsky V.G., Dvoretsky A.G. (2009). Life cycle of *Oithona similis* (Copepoda: Cyclopoida) in Kola Bay (Barents Sea). Mar. Biol..

[60] Dvoretsky V.G., Venger M.P., Vashchenko A.V., Maksimovskaya T.M., Ishkulova T.G., Vodopianova V.V. (2022). Pelagic bacteria and viruses in a high Arctic region: Environmental control in the autumn period. Biology.

[61] Berge J., Renaud P.E., Darnis G., Cottier F., Last K., Gabrielsen T.M., Johnsen G., Seuthe L., Weslawski J.M., Leu E. (2015). In the dark: A review of ecosystem processes during the Arctic polar night. Prog. Oceanogr..

[62] Berge J., Daase M., Hobbs L., Falk-Petersen S., Darnis G., Søreide J.E. (2020). Zooplankton in the polar night. Polar Night Marine Ecology.

[63] Grenvald J.C., Callesen T.A., Daase M., Hobbs L., Darnis G., Renaud P.E., Cottier F., Nielsen T.G., Berge J. (2016). Plankton community composition and vertical migration during polar night in Kongsfjorden. Polar Biol..

[64] Barth-Jensen C., Daase M., Ormańczyk M.R., Varpe, Kwaśniewski S., Svensen C. (2022). High abundances of small copepods early developmental stages and nauplii strengthen the perception of a non-dormant Arctic winter. Polar Biol..

[65] Wietz M., Bienhold C., Metfies K., Torres-Valdés S., von Appen W.J., Salter I., Boetius A. (2021). The polar night shift: Seasonal dynamics and drivers of Arctic Ocean microbiomes revealed by autonomous sampling. ISME Comm..

[66] Parsons T.R., Maita Y., Lalli C.M. (1992). A Manual of Chemical and Biological Methods for Sea Water Analysis.

[67] Aminot A., Rey F. (2000). Standard Procedure for the Determination of Chlorophyll A by Spectroscopic Methods.

[68] Porter K.G., Feig Y.S. (1980). The use of DAPI for identifying and counting aquatic microflora. Limnol. Oceanogr..

[69] Norland S., Kemp P., Sherr B., Sherr E., Cole J. (1993). The relationships between biomass and volume of bacteria. Handbook of Methods in Aquatic Microbial Ecology.

[70] Noble R.T., Fuhrman J.A. (1997). Virus decay and its causes in coastal waters. Appl. Environ. Microbiol..

[71] Dvoretsky V.G., Dvoretsky A.G. (2019). Summer macrozooplankton assemblages of Arctic shelf: A latitudinal study. Cont. Shelf Res..

[72] Chislenko L.L. (1968). Nomogrammes to Determine Weights of Aquatic Organisms Based on the Size and Form of Their Bodies (Marine Mesobenthos and Plankton).

[73] Berestovskij E.G., Anisimova N.A., Denisenk C.G., Luppowa E.N., Savinov V.M., Timofeev S.F. (1989). Relationship between Size and Body Mass of Some Invertebrates and Fish of the North-East Atlantic.

[74] Hanssen H. (1997). Mesozooplankton of the Laptev Sea and the adjacent eastern Nansen Basin–distribution and community structure in late summer. Ber. Polarforsch..

[75] Richter C. (1994). Regional and seasonal variability in the vertical distribution of mesozooplankton in the Greenland Sea. Ber. Polarforsch..

[76] Harris R.P., Wiebe P.H., Lenz J., Skjoldal H.R., Huntley M. (2000). ICES Zooplankton Methodology Manual.

[77] Anderson M.J. (2001). A new method for non-parametric multivariate analysis of variance. Austral Ecol..

[78] Legendre P., Legendre L. (1998). Numerical Ecology.

[79] Hammer, Harper D.A.T., Ryan P.D. (2001). PAST: Paleontological Statistics Software Package for Education and Data Analysis. Palaeontol. Electron..

[80] Shannon C.B., Weaver W. (1963). The Mathematical Theory of Communication.

[81] Pielou E.C. (1966). The measurement of diversity in different types of biological collections. J. Theor. Biol..

[82] Clarke K.R., Warwick R.M. (1994). Changes in Marine Communities: An Approach to Statistical Analysis and Interpretation.

[83] Clarke K.R., Gorley R.N. (2001). PRIMER v5: User Manual/Tutorial.

[84] ter Braak C.J.F., Šmilauer P. (2002). CANOCO Reference Manualand CanoDraw for Windows User’s Guide: Software for Canonical Community Ordination (version 4.5).

[85] Lepš J., Šmilauer P. (2003). Multivariate Analysis of Ecological Data Using CANOCO.

[86] Zar J.H. (1999). Biostatistical Analysis.

[87] McCullagh P., Nelder J.A. (1983). Generalized Linear Models.

[88] Beszczynska-Möller A., Fahrbach E., Schauer U., Hansen E. (2012). Variability in Atlantic water temperature and transport at the entrance to the Arctic Ocean, 1997–2010. ICES J. Mar. Sci..

[89] Tuerena R.E., Hopkins J., Buchanan P.J., Ganeshram R.S., Norman L., von Appen W.J., Mahaffey C. (2021). An Arctic strait of two halves: The changing dynamics of nutrient uptake and limitation across the Fram Strait. Glob. Biogeochem. Cycles.

[90] Tuerena R.E., Mahaffey C., Henley S.F., De La Vega C., Norman L., Brand T., März C. (2022). Nutrient pathways and their susceptibility to past and future change in the Eurasian Arctic Ocean. Ambio.

[91] Wulff T., Bauerfeind E., von Appen W.J. (2016). Physical and ecological processes at a moving ice edge in the Fram Strait as observed with an AUV. Deep-Sea Res. I.

[92] Cardozo-Mino M.G., Fadeev E., Salman-Carvalho V., Boetius A. (2021). Spatial Distribution of Arctic Bacterioplankton Abundance Is Linked to Distinct Water Masses and Summertime Phytoplankton Bloom Dynamics (Fram Strait, 79° N). Front. Microbiol..

[93] Seuthe L., Topper B., Reigstad M., Thyrhaug R., Vaquer-Sunyer R. (2011). Microbial communities and processes in ice-covered Arctic waters of the northwestern Fram Strait (75 to 80 degrees N) during the vernal pre-bloom phase. Aquat. Microb. Ecol..

[94] Rokkan Iversen K., Seuthe L. (2011). Seasonal microbial processes in a high-latitude fjord (Kongsfjorden, Svalbard): I—Heterotrophic bacteria, picoplankton and nanoflagellates. Polar Biol..

[95] Piontek J., Sperling M., Nöthig E.M., Engel A. (2014). Regulation of bacterioplankton activity in Fram Strait (Arctic Ocean) during early summer: The role of organic matter supply and temperature. J. Mar. Syst..

[96] Wilson B., Müller O., Nordmann E.-L., Seuthe L., Bratbak G., Øvreås L. (2017). Changes in Marine Prokaryote Composition with Season and Depth Over an Arctic Polar Year. Front. Mar. Sci..

[97] von Jackowski A., Grosse J., Nöthig E.M., Engel A. (2020). Dynamics of organic matter and bacterial activity in the Fram Strait during summer and autumn. Phil. Trans. R. Soc. A.

[98] von Jackowski A., Becker K.W., Wietz M., Bienhold C., Zäncker B., Nöthig E.M., Engel A. (2022). Variations of microbial communities and substrate regimes in the eastern Fram Strait between summer and fall. Environ. Microbiol..

[99] Venger M.P., Kopylov A.I., Zabotkina E.A., Makarevich P.R. (2016). The influence of viruses on bacterioplankton of the offshore and coastal parts of the Barents Sea. Rus. J. Mar. Biol..

[100] Shirokolobova T.I., Zhichkin A.P., Venger M.P., Vodopyanova V.V., Moiseev D.V. (2016). Bacteria and viruses of the ice-free aquatic area of the Barents sea at the beginning of polar night. Dokl. Biol. Sci..

[101] Sherr E.B., Sherr B.F., Wheeler P.A., Thompson K. (2003). Temporal and spatial variation in stocks of autotrophic and heterotrophic microbes in the upper water column of the central Arctic Ocean. Deep-Sea Res. I.

[102] Maranger R., Vaqué D., Nguyen D., Hébert M.P., Lara E. (2015). Pan-Arctic patterns of planktonic heterotrophic microbial abundance and processes: Controlling factors and potential impacts of warming. Prog. Oceanogr..

[103] Venger M.P., Shirikolobova T.I., Makarevich P.R., Vodopyanova V.V. (2012). Viruses in the pelagic zone of the Barents Sea. Dokl. Biol. Sci..

[104] Błachowiak-Samołyk K., Wiktor J.M., Hegseth E.N., Wold A., Falk-Petersen S., Kubiszyn A.M. (2015). Winter Tales: The dark side of planktonic life. Polar Biol..

[105] Basedow S.L., Sundfjord A., von Appen W.J., Halvorsen E., Kwasniewski S., Reigstad M. (2018). Seasonal Variation in Transport of Zooplankton into the Arctic Basin through the Atlantic Gateway, Fram Strait. Front. Mar. Sci..

[106] Kaiser P., Hagen W., von Appen W.J., Niehoff B., Hildebrandt N., Auel H. (2021). Effects of a Submesoscale Oceanographic Filament on Zooplankton Dynamics in the Arctic Marginal Ice Zone. Front. Mar. Sci..

[107] Svensen C., Seuthe L., Vasilyeva Y., Pasternak A., Hansen E. (2011). Zooplankton distribution across Fram Strait in autumn: Are small copepods and protozooplankton important?. Prog. Oceanogr..

[108] Dvoretsky V.G., Dvoretsky A.G. (2015). Summer population structure of the copepods *Paraeuchaeta* spp. in the Kara Sea. J. Sea Res..

[109] Ducklow H.W., Kirchman D.L. (2000). Bacterial production and biomass in the oceans. Microbial Ecology of the Oceans.

[110] Kirchman D.L., Morán X.A.G., Ducklow H. (2009). Microbial growth in the polar oceans—Role of temperature and potential impact of climate change. Nat. Rev. Microbiol..

[111] Turner J.T. (2004). The importance of small planktonic copepods and their roles in pelagic marine food webs. Zool. Stud..

