# Supplementary material for: Marine Plankton during the Polar Night: Environmental Predictors of Spatial Variability
Source: Biology (Basel). 2023 Feb 25;12(3):368. doi: 10.3390/biology12030368 (PMC10044718; doi:10.3390/biology12030368)
Supplement: Supplementary file 1 [file biology-12-00368-s001.zip › biology-2246835-supplementary.pdf]

Supplementary material

Marine plankton during the polar night: environmental predictors of spatial variability

Vladimir G. Dvoretsky *, Marina P. Venger, Anastasya V. Vashchenko, Veronika V. Vodopianova, Ivan A. Pastukhov and Tatyana M. Maksimovskaya

Biology 2023.

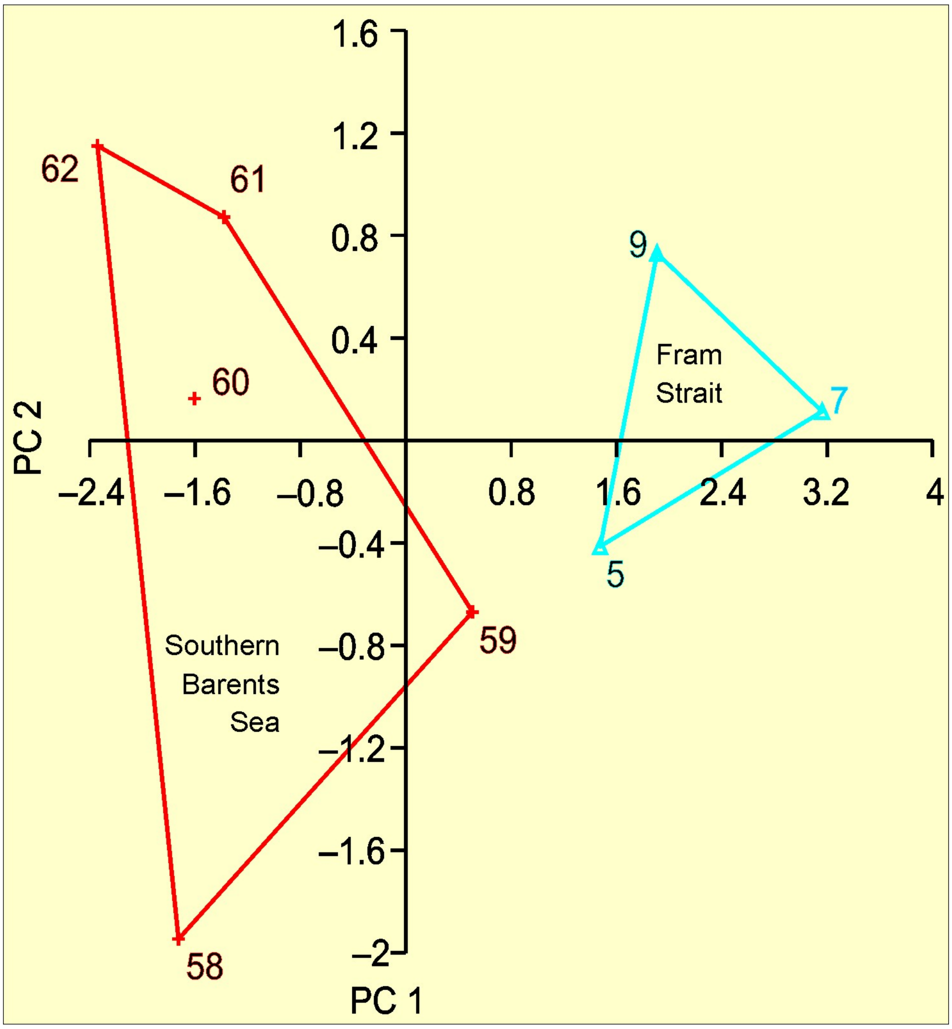

Figure S1. Results of principal component analysis (PCA): ordination diagram indicating separations of sampling stations based on hydrological variables (water temperature and salinity) and hydrochemical parameters (dissolved oxygen, nitrate, phosphate, silicate) in the Barents Sea and Fram Strait, winter 2021.

Table S1. Factor scores extracted with principal component analysis (PCA) based on hydrological variables (water temperature and salinity) and hydrochemical parameters (dissolved oxygen, nitrate, phosphate, silicate) in the Barents Sea and Fram Strait, winter 2021.

Region	Station	PC 1	PC 2	PC 3	PC 4	PC 5	PC 6
Fram Strait	5	1.477	–0.413	1.396	–0.466	0.135	–0.016
Fram Strait	7	3.161	0.113	0.220	0.428	–0.216	–0.019
Fram Strait	9	1.908	0.730	–1.013	–0.580	0.046	0.060
Southern Barents Sea	58	–1.726	–1.946	–0.307	–0.338	–0.108	0.005

Southern Barents Sea	59	0.507	–0.669	–0.588	0.754	0.208	–0.014
Southern Barents Sea	60	–1.605	0.164	0.183	0.280	–0.047	0.058
Southern Barents Sea	61	–1.381	0.872	–0.526	–0.264	–0.013	–0.100
Southern Barents Sea	62	–2.341	1.150	0.635	0.186	–0.005	0.026

Table S2. Factor loadings extracted with principal component analysis (PCA) based on hydrological variables (water temperature and salinity) and hydrochemical parameters (dissolved oxygen, nitrate, phosphate, silicate) in the Barents Sea and Fram Strait, winter 2021.

Factor	PC 1	PC 2	PC 3	PC 4	PC 5	PC 6
Temperature	–0.353	–0.002	0.878	0.316	0.063	0.014
Salinity	0.421	–0.076	0.445	–0.781	0.084	–0.037
Dissolved oxygen	–0.002	0.995	0.028	–0.082	0.019	0.053
Phosphate	0.481	0.025	0.158	0.252	–0.811	0.150
Silicate	0.482	0.063	0.059	0.353	0.271	–0.750
Nitrate	0.484	–0.019	0.037	0.308	0.508	0.641

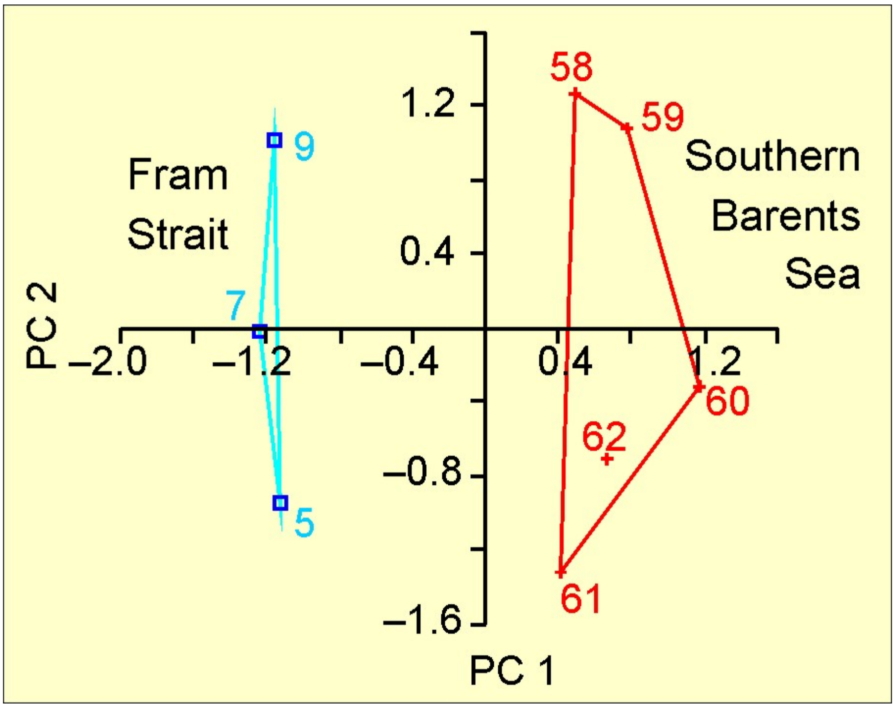

Figure S2. Results of principal component analysis (PCA): ordination diagram indicating separations of sampling stations based on biotic variables (bacterial abundance and biomass, viral abundance, chlorophyll *a* concentration, zooplankton abundance and biomass) in the Barents Sea and Fram Strait, winter 2021.

Table S3. Plankton abundance and biomass (range and mean±SE) in the Fram Strait and in the southern Barents Sea, winter 2021. Comparisons were performed using one-way ANOVA or Kruskal-Wallis test, significant differences at $p<0.05$. n/a - no analysis.

Layer	Fram Strait		Southern Barents Sea		p
		Bacterial abundance, 10^5 cells L^{-1}			
0	3.23–4.23	3.76±0.50	2.26–4.10	3.41±0.78	0.53
10	2.88–4.15	3.33±0.71	2.58–4.30	3.35±0.72	0.98
25	2.88–3.24	3.07±0.18	2.51–6.80	4.00±1.71	0.40
50	2.54–3.48	3.07±0.49	3.54–4.61	4.03±0.54	0.09

100	2.62–3.44	3.07±0.42	2.62–5.25	3.49±1.10	0.56
200	2.86–2.87	2.87±0.01	–	–	n/a
Bottom	2.09–2.86	2.46±0.39	2.30–4.04	2.91±0.67	0.33
0–bottom	2.09–4.23	3.10±0.54	2.26–6.80	3.50±1.01	0.41
		Bacterial biomass, mgC m^{-3}			
0	3.60–3.86	3.74±0.13	3.17–6.26	4.96±1.46	0.22
10	3.19–4.01	3.72±0.46	3.36–5.81	4.50±0.93	0.23
25	2.86–3.31	3.11±0.23	3.22–7.89	5.35±1.67	0.07
50	2.78–3.90	3.36±0.56	3.49–5.63	4.90±1.22	0.12
100	2.96–3.93	3.45±0.49	3.54–6.46	4.50±1.14	0.19
200	2.73–2.79	2.76±0.04	–	–	n/a
Bottom	2.24–3.33	2.61±0.63	3.25–5.45	4.23±0.98	0.04
0–bottom	2.24–4.01	3.27±0.55	3.17–7.89	4.73±1.21	0.02
		Viral abundance, 10^6 particles mL^{-1}			
0	4.03–5.69	4.75±0.86	0.77–2.23	1.52±0.54	0.00
25	3.77–4.13	4.01±0.2	2.29	2.29	n/a
50	3.39–4.8	4.08±0.71	1.2–1.71	1.44±0.26	0.00
100	3.32–4.81	4.16±0.76	1.73–4.85	2.85±1.38	0.14
Bottom	2.04–4.52	3.58±1.34	0.68–1.77	1.14±0.43	0.01
0–bottom	2.04–5.69	3.09±1.96	0.68–4.85	1.74±0.95	0.00
		Chlorophyll *a*, mg m^{-3}			
0	0.01–0.25	0.09±0.14	0.01–0.15	0.09±0.08	0.96
25	0.01–0.08	0.03±0.04	0.01–0.01	0.01±0.00	0.22
50	0.09–0.13	0.11±0.02	0.05–0.09	0.08±0.02	0.11
100	0.01–0.11	0.06±0.05	0.01–0.06	0.03±0.03	0.36
200	0.01–0.01	0.01±0.00	-	-	n/a
Bottom	0.01–0.01	0.01±0.00	0.01–0.01	0.01±0.00	n/a
0–bottom	0.01–0.25	0.05±0.06	0.01–0.15	0.04±0.05	0.41
		Zooplankton abundance, individuals m^{-3}			
0–50	714–1248	919±288	478–753	602±125	0.07
50–bottom	149–316	239±84	369–687	503±148	0.03
0–bottom	149–1248	579±418	369–753	553±139	0.06
		Zooplankton biomass, mgC m^{-3}			
0–50	3.98–14.12	8.08±5.34	0.95–4.70	3.19±1.95	0.10
50–bottom	4.98–10.34	7.75±2.68	1.80–38.74	22.91±14.05	0.12
0–bottom	3.98–14.12	7.91±3.79	0.95–38.74	13.05±14.01	0.03

Table S4. Factor scores extracted with principal component analysis (PCA) based on biotic variables (bacterial abundance and biomass, viral abundance, chlorophyll *a* concentration, zooplankton abundance and biomass) in the Barents Sea and Fram Strait, winter 2021.

Region	Station	PC 1	PC 2	PC 3	PC 4	PC 5	PC 6	PC 7
Fram Strait	5	–1.272	0.901	–1.129	–1.377	–0.219	–0.006	–1.272
Fram Strait	7	–1.013	–0.467	–1.027	1.712	0.640	0.379	–1.013
Fram Strait	9	–1.244	–0.752	1.865	–0.496	–0.054	0.005	–1.244
Southern Barents Sea	58	0.666	–1.346	–0.647	–0.082	–1.666	–0.815	0.666
Southern Barents Sea	59	0.846	–0.960	0.054	–0.368	1.548	0.111	0.846
Southern Barents Sea	60	1.081	0.498	–0.287	–0.835	0.621	0.267	1.081
Southern Barents Sea	61	0.320	1.349	0.619	0.866	0.113	–1.718	0.320
Southern Barents Sea	62	0.615	0.776	0.552	0.580	–0.983	1.777	0.615

Table S5. Factor loadings extracted with principal component analysis (PCA) based on biotic variables (bacterial abundance and biomass, viral abundance, chlorophyll *a* concentration, zooplankton abundance and biomass) in the Barents Sea and Fram Strait, winter 2021.

Factor	PC 1	PC 2	PC 3	PC 4	PC 5	PC 6
Bacterial abundance	0.112	0.656	0.231	0.247	0.084	-0.381
Bacterial biomass	0.344	0.427	0.337	0.191	-0.005	0.241
Viral abundance	-0.453	0.088	0.255	0.320	0.226	0.196
Virus-to-bacteria ratio	-0.465	-0.094	0.186	0.225	0.354	0.366
Average bacterial volume	0.462	-0.113	0.259	-0.063	-0.135	0.663
Chlorophyll *a*	-0.276	0.298	0.319	-0.851	0.066	0.060
Zooplankton abundance	-0.087	-0.408	0.714	0.094	-0.432	-0.339
Zooplankton biomass	0.388	-0.321	0.233	-0.107	0.779	-0.259

Table S6. Results of 18 GLZ models with microplankton characteristics and chlorophyll *a* concentration as the dependent variables and environmental parameters as independent variables in the Fram Strait and in the southern Barents Sea, winter 2021. Only significant explaining variables (p<0.05) are indicated.

Dependent variable	Independent variable	Estimate	SE	Wald statistic	p
All data					
Bacterial abundance	Temperature	–0.066	0.028	5.441	0.020
Bacterial biomass	Salinity	–0.484	0.092	27.865	0.000
Viral abundance	Salinity	1.366	0.549	6.190	0.013
VBR	Salinity	1.392	0.519	7.194	0.007
ABV	Temperature	–0.109	0.053	4.174	0.041
	Salinity	–0.874	0.196	19.887	0.000
Chlorophyll *a*	Temperature	0.674	0.309	4.771	0.029
	Salinity	5.034	1.194	17.761	0.000
	Dissolved oxygen	0.382	0.182	4.385	0.036
	Nitrate	0.012	0.005	7.263	0.007
	Depth	–0.019	0.005	12.656	0.000
Fram Strait					
Bacterial abundance	Depth	–0.001	0.000	5.838	0.016
Bacterial biomass	Depth	–0.001	0.000	18.204	0.000
	Temperature	–0.118	0.053	4.989	0.026
Viral abundance	Depth	–0.028	0.010	7.706	0.006
Chlorophyll *a*	Temperature	2.037	0.626	10.581	0.001
	Nitrate	0.029	0.010	8.509	0.004
Barents Sea					
Bacterial biomass	Salinity	–1.213	0.527	5.3	0.021
Chlorophyll *a*	Dissolved oxygen	0.512	0.258	3.9	0.047

Table S7. Ranking of environmental variables influenced zooplankton assemblages in the Fram Strait and in the southern Barents Sea, winter 2021 (Monte Carlo permutation test, 999 permutations). Significant differences was set at p<0.05.

Variable	Explained variance, %	P	F
Layer	26	0.002	4.95
Temperature	17	0.006	3.92
Salinity	8	0.128	1.82

Nitrate	6	0.142	1.75
Silicate	5	0.361	1.13
Phosphate	4	0.318	1.18
Chlorophyll *a*	4	0.420	1.00
Dissolved oxygen	3	0.600	0.68

Table S8. Results of 24 GLZ models showing interrelations between biotic variables in the Fram Strait and in the southern Barents Sea, winter 2021. Only significant explaining variables ($p<0.05$) are indicated.

Dependent variable	Independent variable	Estimate	SE	Wald statistic	p
	All data				
Bacterial abundance	Bacterial biomass	1.355	0.036	1394	0.000
	ABV	–18.9	0.805	551	0.000
	Chlorophyll *a*	0.120	0.042	8.2	0.004
Bacterial biomass	ABV	19.2	0.543	1244	0.000
Viral abundance	VBR	1.967	0.112	310	0.000
	Chlorophyll *a*	3.147	1.267	6.170	0.013
ABV	Bacterial abundance	–4.083	0.043	8970	0.000
	Bacterial biomass	3.883	0.028	19620	0.000
	Chlorophyll *a*	–0.111	0.034	10.7	0.001
VBR	Viral abundance	2.434	0.229	113	0.000
	Chlorophyll *a*	2.738	1.230	4.951	0.026
Chlorophyll *a*	Bacterial abundance	188.3	40.4	21.8	0.000
	Bacterial biomass	–126.2	23.8	28.1	0.000
	Viral abundance	–52.4	20.5	6.5	0.011
	VBR	51.1	17.3	8.7	0.003
	ABV	2108	384	30.2	0.000
	Zooplankton biomass	–4.179	1.029	16.5	0.000
Zooplankton abundance	Chlorophyll *a*	4.656	2.029	5.3	0.022
Zooplankton biomass	Bacterial abundance	56.2	19.0	8.8	0.003
	ABV	837	253.7	10.9	0.001
	Fram Strait				
Bacterial abundance	Bacterial biomass	1.55	0.05	902	0.000
	Viral abundance	0.12	0.05	4.71	0.030
	VBR	–0.07	0.03	4.81	0.028
	ABV	–24.10	0.86	790	0.000
Bacterial biomass	Viral abundance	1.33	0.23	32.68	0.000
	VBR	–0.83	0.14	33.86	0.000
	ABV	20.04	3.61	30.86	0.000
Viral abundance	VBR	2.05	0.19	120	0.000
	Chlorophyll *a*	6.415	2.560	6.3	0.012
VBR	Chlorophyll *a*	7.255	2.389	9.2	0.002
Chlorophyll *a*	ABV	3016	1337	5.09	0.024
	Bacterial biomass	–99	39	6.33	0.012
Zooplankton abundance	Zooplankton biomass	0.26	0.13	4.05	0.044
	Barents Sea				
Bacterial abundance	Bacterial biomass	1.43	0.04	1234	0.000
	ABV	–18.20	0.67	729	0.000
	Chlorophyll *a*	0.11	0.05	4.32	0.038
Bacterial biomass	ABV	16.69	0.54	967	0.000
	Chlorophyll *a*	0.12	0.05	6.77	0.009
	Bacterial abundance	1.38	0.04	1201	0.000

Viral abundance	VBR	2.31	0.18	166	0.000
Chlorophyll *a*	Bacterial abundance	–241	87.19	7.67	0.006
	VBR	381	171	4.96	0.026
	Bacterial biomass	564	231	5.97	0.015
	Viral abundance	–485	219	4.93	0.026
Zooplankton abundance	Chlorophyll *a*	16.33	3.31	24.38	0.000
	Viral abundance	8.34	3.79	4.84	0.028
Zooplankton biomass	Chlorophyll *a*	32.21	12.41	6.73	0.009
	Bacterial biomass	2628	7.22	132280	0.000
	Viral abundance	234	9.95	552	0.000
